# Role and regulation of coordinately expressed *de novo* purine biosynthetic enzymes *PPAT* and *PAICS* in lung cancer

**DOI:** 10.18632/oncotarget.4352

**Published:** 2015-06-23

**Authors:** Moloy T. Goswami, Guoan Chen, Balabhadrapatruni V.S.K. Chakravarthi, Satya S. Pathi, Sharath K. Anand, Shannon L. Carskadon, Thomas J. Giordano, Arul M. Chinnaiyan, Dafydd G. Thomas, Nallasivam Palanisamy, David G. Beer, Sooryanarayana Varambally

**Affiliations:** ^1^ Michigan Center for Translational Pathology, Ann Arbor, MI 48109, USA; ^2^ Department of Pathology, University of Michigan, Ann Arbor, MI 48109, USA; ^3^ Department of Urology, University of Michigan, Ann Arbor, MI 48109, USA; ^4^ Thoracic Surgery, Department of Surgery, University of Michigan, Ann Arbor, MI 48109, USA; ^5^ Comprehensive Cancer Center, University of Michigan Medical School, Ann Arbor, MI 48109, USA; ^6^ Howard Hughes Medical Institute, University of Michigan Medical School, Ann Arbor, MI 48109, USA; ^7^ Department of Urology, Henry Ford Health System, Detroit, MI 48202, USA; ^8^ Department of Pathology, University of Alabama at Birmingham, Birmingham, AL 35233, USA; ^9^ Immunobiology and Cancer Research Program, Oklahoma Medical Research Foundation, Oklahoma City, OK 73104, USA

**Keywords:** lung adenocarcinoma, amplification, purine biosynthesis, glutamine, xenograft

## Abstract

Cancer cells exhibit altered metabolism including aerobic glycolysis that channels several glycolytic intermediates into *de novo* purine biosynthetic pathway. We discovered increased expression of phosphoribosyl amidotransferase (PPAT) and phosphoribosylaminoimidazole carboxylase, phosphoribosylaminoimidazole succinocarboxamide synthetase (PAICS) enzymes of *de novo* purine biosynthetic pathway in lung adenocarcinomas. Transcript analyses from next-generation RNA sequencing and gene expression profiling studies suggested that PPAT and PAICS can serve as prognostic biomarkers for aggressive lung adenocarcinoma. Immunohistochemical analysis of PAICS performed on tissue microarrays showed increased expression with disease progression and was significantly associated with poor prognosis. Through gene knockdown and over-expression studies we demonstrate that altering PPAT and PAICS expression modulates pyruvate kinase activity, cell proliferation and invasion. Furthermore we identified genomic amplification and aneuploidy of the divergently transcribed PPAT-PAICS genomic region in a subset of lung cancers. We also present evidence for regulation of both PPAT and PAICS and pyruvate kinase activity by L-glutamine, a co-substrate for PPAT. A glutamine antagonist, 6-Diazo-5-oxo-L-norleucine (DON) blocked glutamine mediated induction of PPAT and PAICS as well as reduced pyruvate kinase activity. In summary, this study reveals the regulatory mechanisms by which purine biosynthetic pathway enzymes PPAT and PAICS, and pyruvate kinase activity is increased and exposes an existing metabolic vulnerability in lung cancer cells that can be explored for pharmacological intervention.

## INTRODUCTION

Lung cancer is the leading cause of cancer-related deaths globally [1] with non-small cell lung cancer (NSCLC) accounting for 80% of all lung cancers [2, 3]. In the United States, the overall 5-year survival rate of lung cancer patients is 16% and more than 50% of the cases are diagnosed at advanced stages where curative treatment is not possible. Genomic and gene expression-based characterizations have resulted in improved molecular subtyping of lung cancer and identification of driver mutations including epidermal growth factor receptor (*EGFR*-10–30%), Kirsten rat sarcoma viral oncogene homolog (*KRAS*-15–30%), and fibroblast growth factor receptor 1 (*FGFR1*-20%), among others [4, 5]. Identification of *EGFR* activating mutations in its kinase domain led to the development of EGFR tyrosine kinase inhibitors, erlotinib and gefitinib [5, 6]. Lung cancer patients harboring *ALK* gene fusions treated with crizotinib show similar positive response [7, 8]. In recent studies, identification of fusions of *CD74-NRG1* that activate HER2:HER3 signaling offered therapeutic possibilities for intractable mucinous lung cancers [9, 10]. Thus, well-defined molecular stratification has become essential for the development of targeted treatment for specific molecular subtypes of cancers and personalized therapy.

Recent studies have identified various metabolic genes and associated metabolites that play a role in oncogenesis. For example, *IDH1* and *IDH2* mutations confer altered enzyme properties producing D-2-hydroxyglutarate and triggering oncogenesis [11, 12]. Our earlier studies revealed that the metabolite sarcosine and its biosynthetic enzyme, glycine N-methyltransferase (GNMT) were elevated in prostate cancer, while sarcosine dehydrogenase (SARDH) and pipecolic acid oxidase (PIPOX) that metabolize sarcosine, were reduced in prostate tumors [13, 14]. In addition, *KRAS*-mediated metabolic transformation is known to activate the phosphate pentose pathway and hexosamine biosynthesis in pancreatic cancer [15]. Various oncogenes such as MYC have been shown to induce purine and pyrimidine biosynthesis genes including phosphoribosyl amidotransferase (*PPAT*), phosphoribosylaminoimidazole carboxylase, phosphoribosylaminoimidazole succinocarboxamide synthetase (*PAICS*), adenylosuccinate Lyase (*ADSL*), dihydroorotate dehydrogenase (*DHODH*) and others [16, 17]. In addition, a yeast screen identified the purine biosynthetic gene *PAICS* as an anti-apoptotic oncogene [18]. Aerobic glycolysis, also known as the Warburg effect, is a well-known metabolic aberration occurring during oncogenesis [19, 20] that allows cancer cells to siphon glycolytic intermediates into anabolic pathways such as purine and pyrimidine biosynthesis to sustain increased cell proliferation [21]. These studies have advanced our understanding of driver genes and their effect on metabolic pathways involved in cancer and underscore the importance of metabolic dysregulation in cancer progression [22].

Purine nucleotides are synthesized through two distinct pathways, either *de novo* utilizing simple precursors like amino acids (glutamine, glycine and aspartate) and bicarbonate or through salvage of purine bases released by the hydrolytic degradation of nucleic acids and nucleotides. *De novo* purine biosynthesis requires 5-phosphoribosyl-1-pyrophosphate (PRPP) as a precursor and glutamine as co-substrate [23]. The initial committed step in this pathway is catalyzed by *PPAT* leading to production of 5-phosphoribosyl-1-amine (5-PRA). *PPAT* is divergently transcribed from a locus that also encodes *PAICS*, another enzyme in the pathway that produces the intermediary metabolite N-succinyl-5-aminoimidazole-4-carboxamide-1-ribose-5′-phosphate (SAICAR), known to activate pyruvate kinase isoform *PKM2* under glucose-depleted condition [24]. After the initial committed step, in a series of enzymatic reactions, 5-PRA is converted to inosine monophosphate (IMP), the precursor for nucleotides adenosine monophosphate (AMP) and guanosine monophosphate (GMP).

In this study, we demonstrate that *de novo* purine biosynthetic pathway enzymes *PPAT* and *PAICS* show increased expression in lung cancer and can serve as prognostic markers for patient survival. Further, our investigations indicate *PPAT* and *PAICS* genes are necessary for lung tumorigenesis. We show that *PPAT* and *PAICS* expression influences the pyruvate kinase activity. Finally, our results reveal glutamine mediated modulation govern *PPAT* and *PAICS* over-expression.

## RESULTS

### *De novo* purine biosynthetic pathway genes are overexpressed in lung cancers

The *de novo* purine biosynthetic pathway involves multiple enzymatic steps that convert 5-phosphoribosyl-1-pyrophosphate (PRPP) to inosine monophosphate (IMP), a precursor for adenosine monophosphate (AMP) and guanosine monophosphate (GMP) production. Many of the bifunctional enzymes catalyze more than one step in the pathway. Recent studies have suggested that N-succinyl-5-aminoimidazole-4-carboxamide-1-ribose-5′-phosphate (SAICAR), a product of the enzyme PAICS, is involved in activating pyruvate kinase PKM2 under glucose-depleting condition [24]. PKM2 is involved in increased aerobic glycolysis in cancer [25], and plays a non-metabolic role in cancer by modifying histone H3 [26].

**Figure 1:**
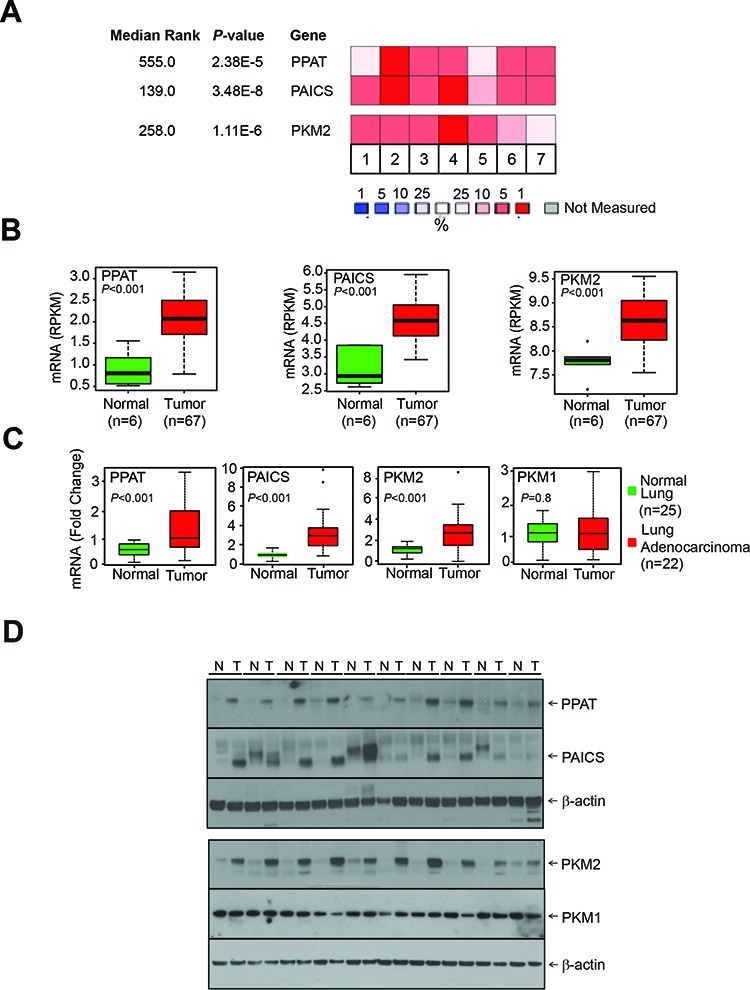
*De novo* purine biosynthetic enzymes, *PPAT & PAICS* and pyruvate kinase isoform *PKM2* are overexpressed in lung adenocarcinoma **A.** Gene expression profiling [28, 30, 32, 33, 73–75] studies suggest enhanced expression of *de novo* purine biosynthetic enzymes *PPAT* & *PAICS* and *PKM2* in multiple lung adenocarcinoma tissues. Expression is represented by a color scale highlighting down-regulation (blue), no alteration (white), and up-regulation (red) of transcripts. **B.** Quantitative measurement of *PPAT*, *PAICS* and *PKM2* by Next-Generation RNA sequencing [RPKM (log2)] in lung adenocarcinomas [39]. **C.** Quantitative real-time polymerase chain reaction (qRT-PCR) measurement of *PPAT*, *PAICS*, *PKM2* and *PKM1* transcripts in lung adenocarcinomas. *GAPDH* transcript levels was used as control. **D.** Immunoblot analysis of lung adenocarcinoma tissue lysates (T) and matched normal samples (N) confirm increased expression of *de novo* purine biosynthetic enzymes *PPAT*, *PAICS* as well as specific PKM isoform *PKM2* in lung cancer. *PKM1* isoform expression was tested and did not show difference between normal and cancer tissues. β-actin was used as a loading control.

Our analysis using lung cancer gene expression profiling studies [27–33] available in the Oncomine [Oncomine™ Platform (Life Technologies, Ann Arbor, MI) was used for analysis and visualization] [34], showed significantly increased expression of *de novo* purine biosynthetic enzymes phosphoribosyl pyrophosphate amidotransferase (*PPAT*), phosphoribosylaminoimidazole carboxylase, phosphoribosylaminoimidazole succinocarboxamide synthetase (*PAICS*) (Fig. 1A; Supplementary Fig. S1A). The pyruvate kinase isoform PKM2 expression also showed increased expression in lung cancer. PPAT is involved in conversion of phosphoribosyl pyrophosphate (PRPP) to 5-phosphoribosylamine (5-PRA), a committing step in *de novo* purine synthesis. We further confirmed the increased expression of divergently transcribed *PPAT*, *PAICS* as well as *PKM2* expression in lung cancer samples compared to normal samples by next-generation RNA sequencing (Fig. 1B) and quantitative RT-PCR analysis (Fig. 1C). We found no difference in *PKM1* transcript expression (Fig. 1C). In addition, Affymetrix microarray analyses showed enhanced expression of *PPAT*, *PAICS* and *PKM2* in poorly differentiated and stage 3 lung cancers (Supplementary Fig. S2A and S2B, respectively). Furthermore, our analysis showed that *PPAT* and *PAICS* are highly expressed in the solid type of adenocarcinoma (Supplementary Fig. S2C) [35]. Analyses of other genes in *de novo* purine biosynthetic pathway enzymes (purinosome) by oncomine (Supplementary Fig. S1A) [33], gene expression from Seo *et al* [36] and TCGA datasets [37, 38] (Supplementary Fig. S1B), and next-generation RNA sequencing (Supplementary Fig. S1C) showed increased expression of glycinamide ribonucleotide formyltransferase (a trifunctional enzyme with GARS, AIRS, GART activity) (*GART*), 5-aminoimidazole-4-carboxamide ribonucleotide formyltransferase/IMP cyclohydrolase (*ATIC*) and phosphoribosylformylglycinamidine synthase (*PFAS)*, but not adenylosuccinate lyase (*ADSL*) levels (Supplementary Fig. S1C) [39]. Consistent with RNA expression, immunoblot analyses of PPAT, PAICS and PKM2 showed increased expression in cancer tissues compared to normal lung (Fig. 1D). However, PKM1 protein did not show increased expression in cancer similar to the transcript data (Fig. 1D).

### *PPAT* and *PAICS* overexpression predicts poor survival in lung cancer

**Figure 2:**
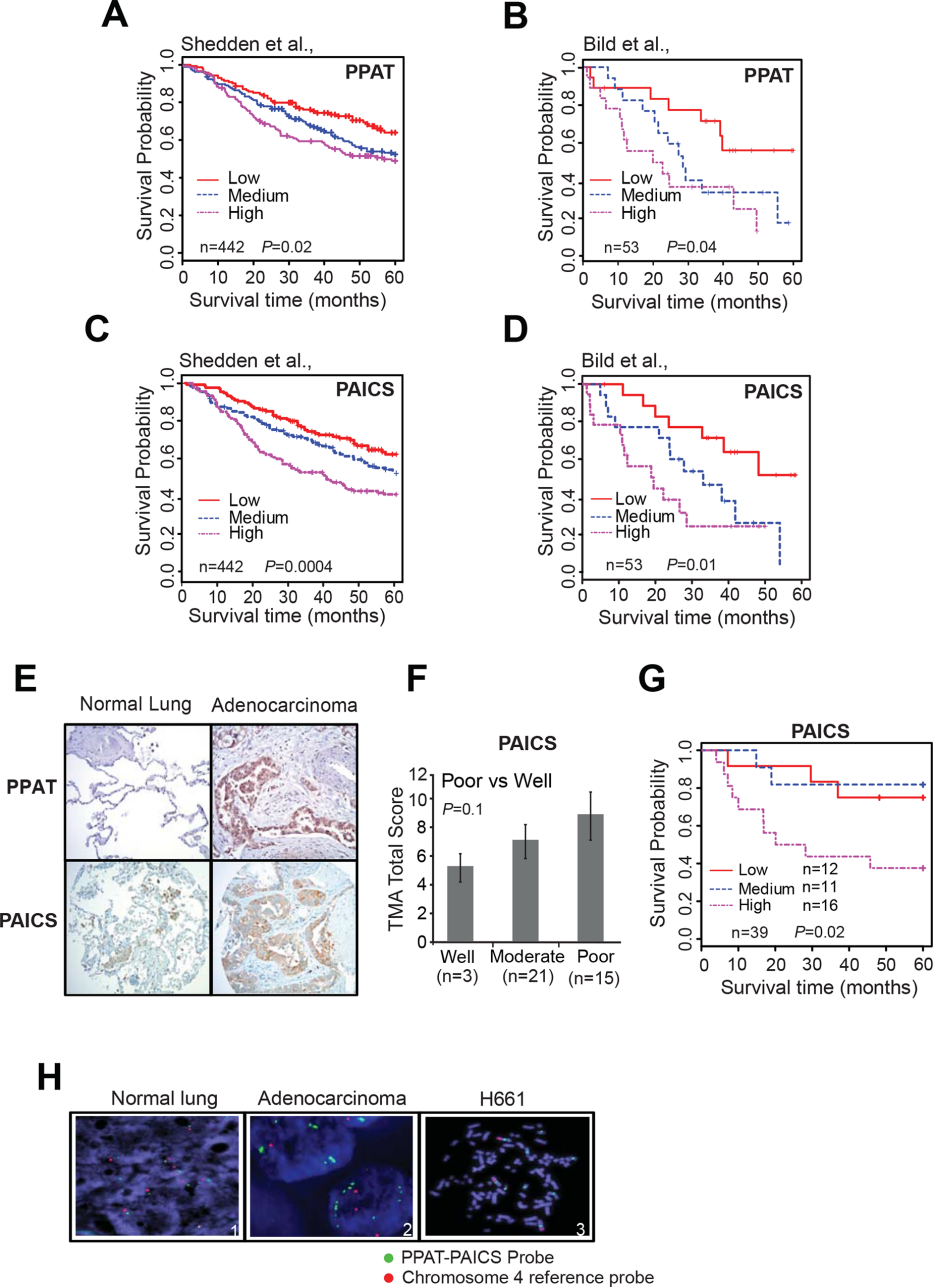
Increased expression of *PPAT* and *PAICS* correlates with aggressive lung cancer and is amplified in a subset of lung cancer **A–D.** Kaplan-Meier (K-M) analysis of survival time according to the *PPAT* and *PAICS* transcript levels as measured using Affymetrix oligonucleotide microarray datasets by Shedden *et al*., [35] Bild *et al*. [40], respectively. The CEL files of microarray data were normalized using Robust Multi-array Average (RMA) method. Expression was classified into low, medium and high expressing groups. Five year survival time was used for K-M calculations. **E.** Photomicrographs of *PPAT* and *PAICS* immunostaining in normal lung (left) and lung adenocarcinoma tissues (right) using PPAT and PAICS-specific antibodies. **F.** Tumors are classified into well differentiated, moderately differentiated and poorly differentiated class based on the total score of immunostaining. **G.** The immunohistochemistry on a lung adenocarcinoma tissue microarray was scored and K-M survival curve was calculated based on staining intensity. Like transcript levels, protein expression was also sub-grouped into three classes. **H.** FISH was performed in normal and lung tumor tissues (Adenocarcinomas) and H661 (large cell carcinoma).

Kaplan-Meier analysis using transcript data for *PPAT* (Fig. 2A and 2B; Supplementary Fig. S2D) and *PAICS* revealed (Fig. 2C and 2D; Supplementary Fig. S2D) that they predict poor patient overall survival (OS) and disease free survival (DFS) with increased expression of these genes in two independent studies [35, 40]. Similarly, elevated *PKM2* expression was also correlated with poor patient survival (Supplementary Fig. S3A) [35, 40]. Multivariate Cox model analysis (with gene, age, gender, stage and differentiation in the model) indicated that *PAICS*, *PPAT* and *PKM2* are also independently associated with patient survival in lung adenocarcinomas [HR 1.31 (1.05–1.64, *P* = 0.02), 1.41 (1.03–1.92, *P* = 0.03), 1.60 (1.18–2.17, *P* = 0.002) for *PAICS*, *PPAT* or *PKM2* respectively]. Thus, our observations demonstrate that expression of *PPAT*, *PAICS* as well as *PKM2* serve as biomarkers of disease progression. Tissue microarray analysis by immunohistochemistry (IHC) using specific antibodies against *PPAT* and *PAICS* showed increased staining in lung adenocarcinomas (Fig. 2E). Furthermore, increased expression of PAICS is associated with poorly differentiated and more aggressive tumors (Fig. 2F). Kaplan-Meier analysis of those samples also revealed that increased PAICS protein expression related to poor patient survival (Fig. 2G) corroborating transcript (RNA) and protein data. In addition, PKM2 showed increased expression in adenocarcinomas (Supplementary Fig. S3B). Among the lung adenocarcinoma patients, smokers (both past and current) showed elevated *PPAT* and *PAICS* expression than non-smokers while *PKM2* expression levels were elevated in lung adenocarcinoma patients above age 60 (Supplementary Table S1). In order to identify the possible genomic events for PPAT and PAICS dysregulation, we performed fluorescence *in situ* hybridization (FISH) with lung adenocarcinoma samples. We discovered aneuploidy and amplification in the divergently transcribed locus of *PPAT* and *PAICS* in a subset of lung adenocarcinoma (∼3% cases) and amplification in H661 lung cancer cell line (Fig. 2H). *PPAT* and *PAICS* are located within the 4q12 chromosomal segment that earlier has been reported to be amplified in NSCLC [41]. Thus amplification could be one of the potential mechanisms of increased expression of PPAT and PAICS in a subset of lung adenocarcinomas.

### *PPAT*, *PAICS* and *PKM2* are critical for lung cancer cell proliferation and invasion

**Figure 3:**
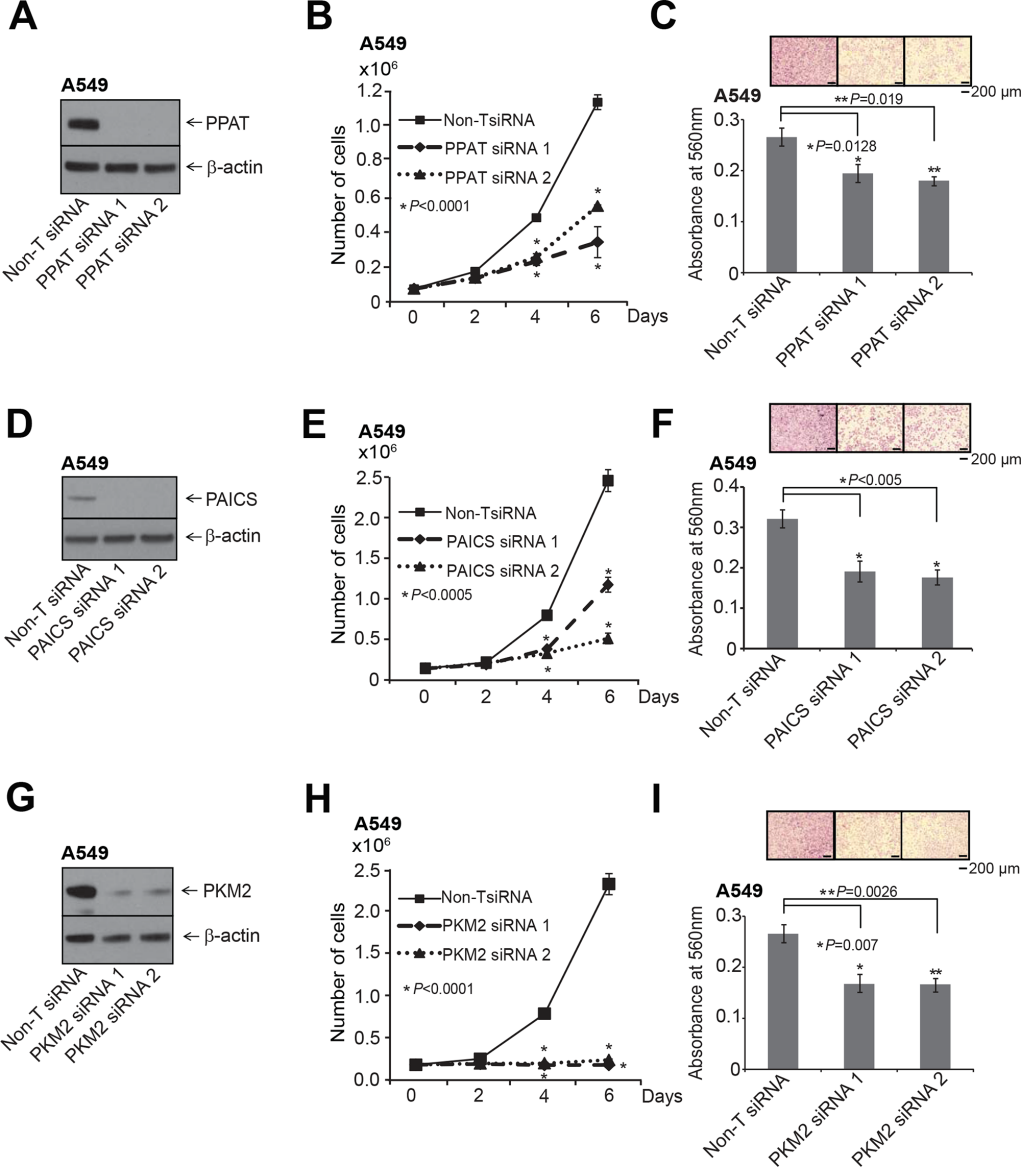
*PPAT, PAICS* and *PKM2* regulate cell proliferation and invasion in A549 lung adenocarcinoma cell lines The knockdown of each gene was achieved using two independent siRNAs. **A.** *PPAT* gene specific, **D.** *PAICS* gene specific and **G.** *PKM2* isoform specific siRNAs were used for the knockdown. **A, D.** and **G.** show immunoblot analysis to verify knockdown of respective genes. **B, E.** and **H.** *PPAT*, *PAICS* and *PKM2* knockdown in A549 cells exhibit reduced cell proliferation. Asterisk indicates data is statistically significant (*P* < 0.05). The solid black line is for Non-specific target (Non-T) siRNA, the dashed line is for siRNA1, and dotted line is for siRNA2. **C, F.** and **I.** represent Boyden chamber matrigel invasion assay. Photomicrographs of invaded cells (purple) are shown in inset. For proliferation and invasion experiments, mean (*n* = 3) +/− SD is shown. Values with *P* <0.05 has been considered significant and marked with asterisk (*).

Normal cells fulfill their purine and pyrimidine requirements through the salvage pathway. Highly proliferative and cancer cells are speculated to meet these requirements by activation of the *de novo* biosynthetic pathway [42]. We investigated the functional significance of the *de novo* purine biosynthetic pathway genes PPAT and PAICS in lung cancer by loss-of-function analyses of these divergently transcribed and elevated in lung tumors. Additionally, we evaluated the functional role of isoform of pyruvate kinase PKM2 in lung carcinomas since they were known to be activated in glucose depleted condition by the PAICS metabolite SAICAR. We performed knockdown experiments using two independent gene-specific siRNAs. Of note we custom synthesized siRNA duplexes for the PKM2 isoform [43]. Knockdown efficiency was confirmed by immunoblot analysis using specific antibodies (Fig. 3A, 3D and 3G) and by qRT-PCR (Supplementary Fig. S4A–S4C). Knockdown of each of these enzymes resulted in significant decrease in cell proliferation (Fig. 3B, 3E and 3H) and reduced cancer cell invasion as measured by Boyden chamber matrigel invasion assays (Fig. 3C, 3F and 3I), implicating a role for these genes in cancer cell growth and invasion. Our observation that PAICS knockdown inhibits cell proliferation concurs with similar observations in melanoma cells [18]. Cell cycle analyses revealed (Supplementary Fig. S5A and S5B) increase in S-phase population in the *PPAT* and *PAICS* knockdowns compared to controls. This observation is similar and consistent with slowing down of cell cycle when purine biosynthesis is blocked using anti-folates [44]. In addition, we found reduction in cell proliferation in H661, a large cell lung carcinoma cell line (Supplementary Fig. S6A–S6C) that harbors *PPAT* and *PAICS* gene amplification (Fig. 2H) upon knockdown of *PPAT*, *PAICS* and *PKM2*. Knockdown efficiency was confirmed by immunoblot analysis using specific antibodies (Supplementary Fig. S6A–S6C, insets) and by qRT-PCR (Supplementary Fig. S4D–S4F). These *in vitro* experiments underscore role of PPAT, PAICS and PKM2 in proliferation and invasion across a cross-section of lung cancers cells.

### *PPAT* and *PAICS* expression modulate pyruvate kinase activity

**Figure 4:**
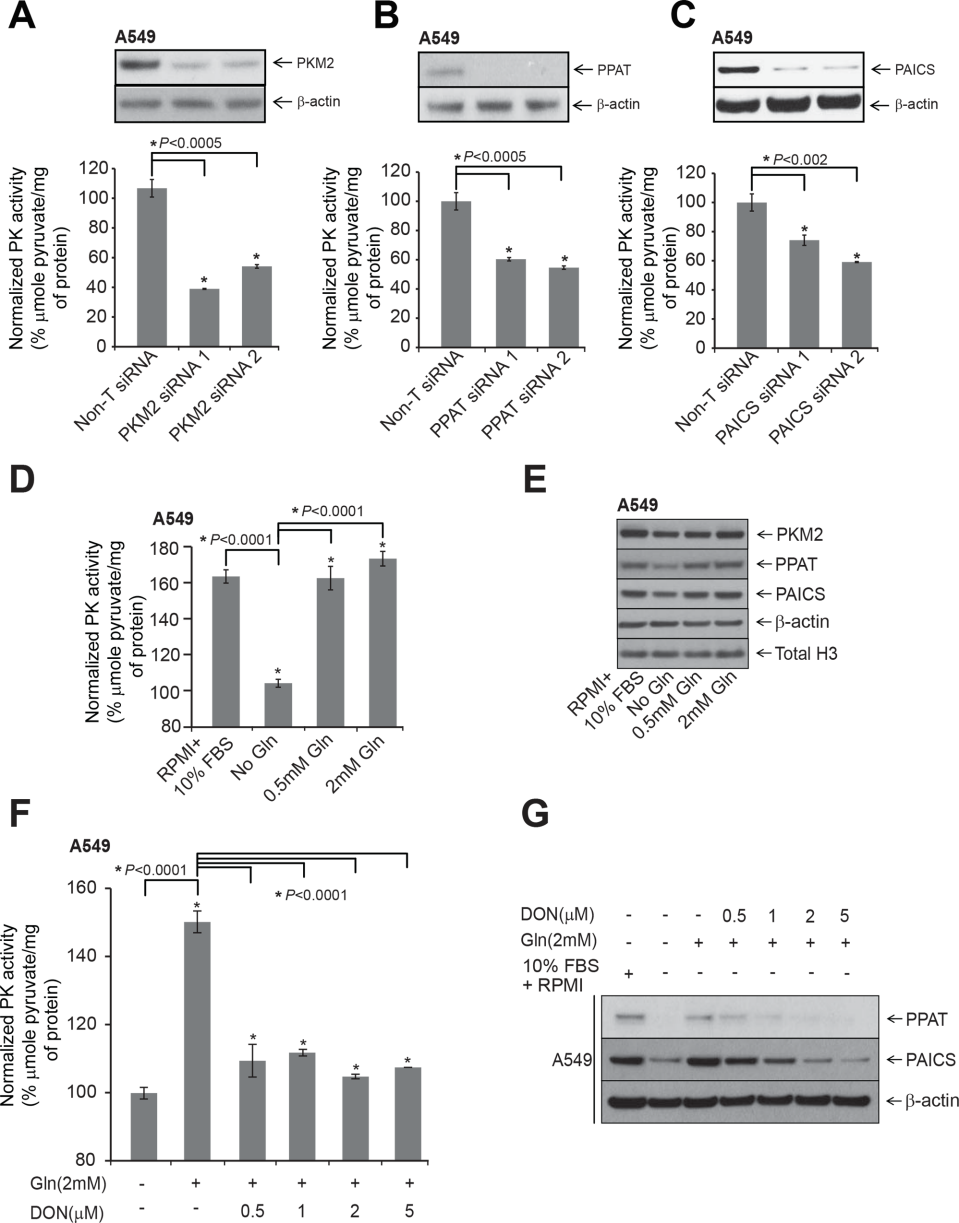
Modulation of *PPAT* and *PAICS* or glutamine treatment alters pyruvate kinase (PK) activity **A, B.** and **C.** PK activity measured in *PKM2*, *PPAT* and *PAICS* knockdown A549 lung adenocarcinoma cell lines. Inset shows immunoblot confirming specific knockdown of the respective genes. **D.** Measurement of PK activity in A549 cells following treatment with RPMI+10% FBS, no glutamine and glutamine (0.5 mM and 2 mM) treatments. **E.** Immunoblot analyses of *PPAT*, *PAICS* and *PKM2* following various treatments for 48 hrs. β-actin and total H3 were used as loading controls. **F.** Measurement of PK activity in A549 cells following treatment with glutamine (2 mM) and increasing concentration of DON after a period of 48 hrs. **G.** Immunoblot analyses with *PPAT* and *PAICS* in A549 cells following treatment with glutamine (2 mM) or increasing concentration of DON after 48 hrs. Minus glutamine has been considered as negative controls and β-actin has been used as loading control.

Based on a published report that PAICS product, SAICAR acts as an allosteric activator of PKM2 under glucose depleted conditions [24], we hypothesized that apart from *PAICS*, *PPAT* expression also potentially can regulate pyruvate kinase activity. We first knocked down PKM2 and tested for Pyruvate Kinase (PK) activity in A549 lung adenocarcinoma cells. We observed a decrease in PK activity upon PKM2-targeted knockdown in normal medium compared to Non-T siRNA (Fig. 4A). Moreover *PPAT* and *PAICS* knockdowns also showed significant decrease in PK activity (Fig. 4B and 4C, respectively). A similar decrease in PK activity was observed with PAICS (Supplementary Fig. S7A and S7B) and PKM2 (Supplementary Fig. S7C and S7D) knockdowns in H661 cells. Furthermore, our data shows a role for *PPAT*, an enzyme upstream of PAICS in *de novo* purine biosynthesis pathway in regulating PK activity.

### Glutamine regulates PK activity, induces *PPAT* and *PAICS* expression

Glutamine plays a critical role in cancer metabolism, [45, 46] and serves as a co-substrate for PPAT and PFAS. The final step of *de novo* pathway in which xanthine 5′-monophosphate is converted to guanosine 5′-monophosphate (GMP) also requires glutamine as amino donor [23]. We therefore tested the effect of glutamine both in regulation of PK activity as well *PPAT* and *PAICS* expression. Glutamine deprivation dramatically reduced A549 cell proliferation (Supplementary Fig. S8A) as observed previously [47]. Glutamine deprivation significantly reduced PK activity (Fig. 4D) and moderately reduced PKM2 protein expression (Fig. 4E). Treatment with similar concentrations of alanine failed to change PK activity over no glutamine controls (Supplementary Fig. S8B). In addition, pyruvate kinase activity and reduction in expression of *PPAT* and *PAICS* by glutamine deprivation could be entirely restored by exogenous addition of glutamine in A549 cells (Fig. 4D and 4E, respectively). We found similar, albeit less dramatic effects of glutamine deprivation on cell proliferation of H661 and H838 cells (Supplementary Fig. S8C and S8E, respectively), and *PPAT* and *PAICS* expression (Supplementary Fig. S8D and S8F), suggesting one of several critical roles of glutamine is in activating *de novo* purine biosynthesis pathway genes and altering cancer cell metabolism. Furthermore, treating A549 cells with increasing concentration of glutamine antagonist, L-diazo-5-oxo-L-norleucine (DON) [48] reduced PK activity (Fig. 4F) and *PPAT* and *PAICS* expression (Fig. 4G) even in presence of glutamine that were comparable to glutamine starved controls. Conversely, over-expression of *PAICS* in the benign lung cells, BEAS-2B resulted in increased PK activity (Supplementary Fig. 9A) and invasion (Supplementary Fig. 9B). Taken together, these results demonstrate that glutamine activates *de novo* purine biosynthetic pathway genes *PPAT* and *PAICS* and increases pyruvate kinase activity.

### *PPAT* and *PAICS* play a role in lung tumor growth

**Figure 5:**
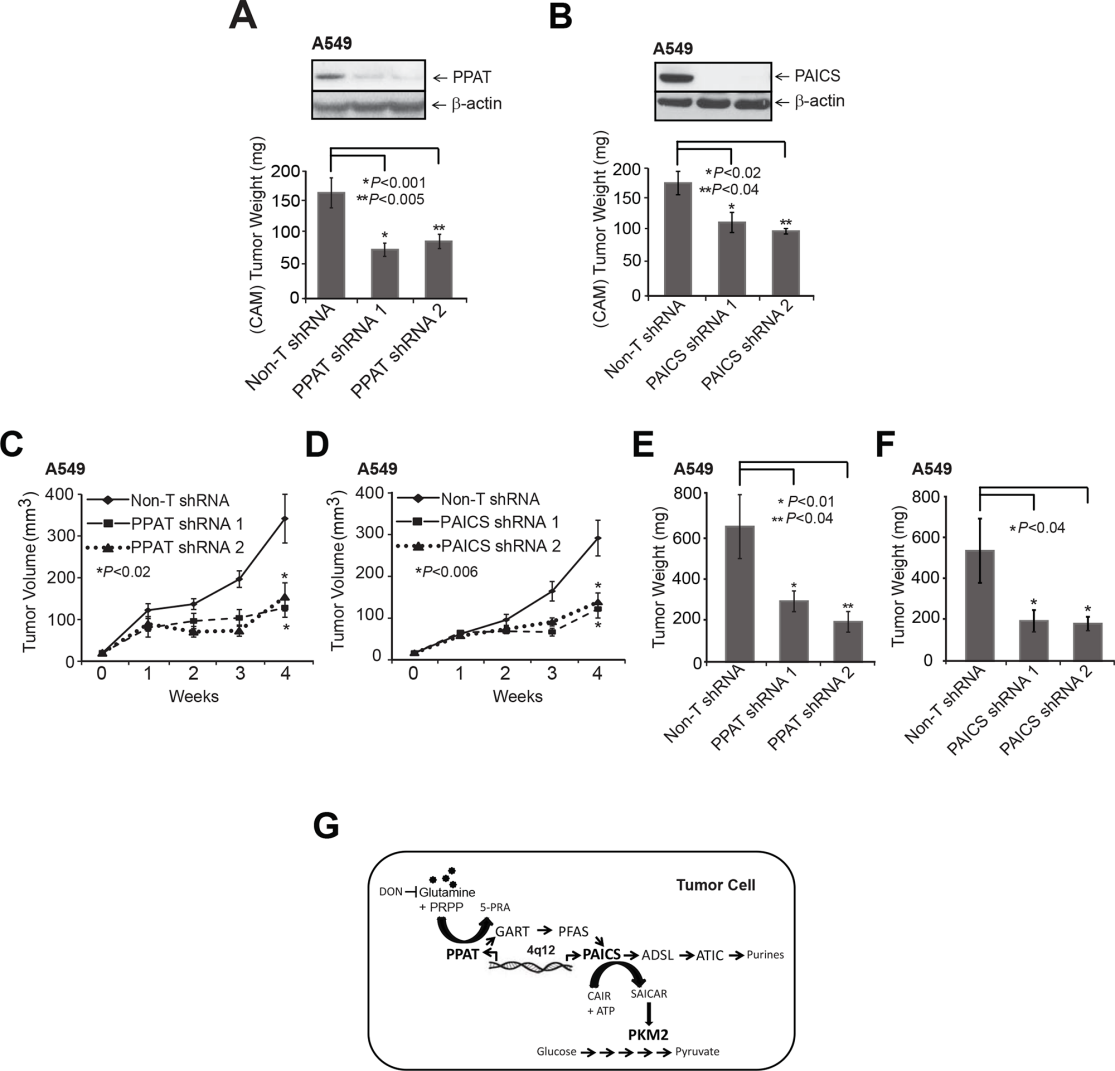
Stable knockdown of *PPAT* and *PAICS* reduces tumor growth in xenograft models **A.** *PPAT* and **B.** *PAICS* knockdown using two independent shRNAs were utilized in the *in vivo* chicken chorioallantoic membrane (CAM) assay. Tumor growth was measured in *PPAT* and *PAICS* stable knockdown cells as well as in control A549 non-targeting shRNA cells (adenocarcinoma). Tumor size plotted correspond to average tumor size of 8 eggs per group and +/− SE per group (*n* = 8). **C–F.** Athymic nude mice were injected with A549 cells that had stable *PPAT* and *PAICS* knockdown separately and tumors were monitored over four weeks (C and D respectively); following which the mice were sacrificed and tumor weights were measured (E and F respectively). Non-targeting shRNA was used as control. The solid black line is for Non-T shRNA, the dashed line is for shRNA1 and dotted line is for shRNA2. Each value is tumor mean (*n* = 16) +/−SE from 8 mice; *P* <0.05 is considered as significant. **G.** Our study showed increased expression of *de novo* purine biosynthesis pathway genes PPAT and PAICS in lung cancer, their regulation by amplification and through induction by glutamine. This study also shows a role for PPAT and PAICS in lung tumorigenesis. Glutamine analog DON blocks the glutamine mediated induction of *PPAT* and *PAICS*. Increased expression of *PPAT* and *PAICS* modulates PK activity possibly by influencing levels of SAICAR [24], the product of PAICS in cancer cells. These enzymes therefore serve as effective therapeutic targets.

Extending the *in vitro* studies described above, we next tested the role of *PPAT* and *PAICS* in two *in vivo* lung cancer xenograft models. We generated two independent stable knockdowns of *PPAT* and *PAICS* in A549 cells (Fig. 5A and 5B, inset) and used them in a chicken chorioallantoic membrane (CAM) assay that has been successfully used as an *in vivo* model to faithfully recapitulate several features of oncogenesis including tumor growth, local invasion and metastasis [49–51]. A549 cells with stable knockdown of *PPAT* or *PAICS* showed reduction in CAM tumor growth compared to cells with non-targeting shRNA controls (Fig. 5A and 5B, respectively). Similarly, H23 (lung adenocarcinoma cells) with PAICS knockdown (Supplementary Fig. S10A) showed reduced cell proliferation (Supplementary Fig. S10B) and a decrease in CAM tumor growth (Supplementary Fig. S10C). Next, we performed mouse xenograft experiments where athymic nude mice were injected subcutaneously with *PPAT* and *PAICS* stable knockdown A549 cells and tumors were subsequently measured. A significant reduction both in tumor growth and weight was observed in both *PPAT* (Fig. 5C and 5E, respectively) and *PAICS* (Fig. 5D and 5F, respectively) knockdowns compared to the control cells, demonstrating that *PPAT* and *PAICS* play essential role in lung tumor growth *in vivo*.

Our data demonstrate (Fig. 5G) that PPAT and PAICS of *de novo* purine biosynthesis show increased expression in lung cancer compared to normal lung. *PPAT* and *PAICS* are two such genes that are co-transcribed from locus 4q12 and show genomic amplification in small percentage of lung cancer cases. Furthermore, our studies show that *PPAT* and *PAICS* play an oncogenic role in lung tumorigenesis. In addition, we show that *PPAT* and *PAICS* expression levels and pyruvate kinase activity are modulated by glutamine. Finally, *PPAT* and *PAICS* [24] levels regulate pyruvate kinase activity that is comparable to *PKM2* depletion. This could possibly be through modulating levels of SAICAR, the PAICS enzyme product that directly interacts and activate *PKM2* activity [24]. Further studies are needed to understand the exact mechanisms by which *PPAT* and *PAICS* modulate PK activity in normal glucose medium.

## DISCUSSION

Recent reports from the World Health Organization warns of a dramatic global rise in cancer incidence and related mortality [52]. Lung cancer, which accounts for the most cancer-related deaths, is a heterogeneous disease and a leading cause of cancer-related mortality in the United States [1]. Non-small cell lung cancer (NSCLC) accounts for 90% of lung cancer while small-cell lung cancer (SCLC) accounts for the remainder [6]. Advancement in whole genome, exome and transcriptome sequencing technology has led to the stratification of lung cancer into specific molecular subtypes based on mutations they harbor [5]. For example, as discussed earlier, mutation in *EGFR* accounts for 10–30%, *KRAS* for 15–30%, and *FGFR1* for 20% of NSCLC and targeted therapies against specific mutations has proven clinically more beneficial to patients compared to the cytotoxic chemotherapies [4]. Unfortunately, >50% of NSCLC harbor no targetable oncogenic driver mutations and many are usually diagnosed at an advanced stage. The intractable nature of lung cancer necessitates search for early diagnostic markers and identification of relevant therapeutic targets to improve patient prognosis. We showed that PPAT and PAICS of *de novo* purine biosynthesis are up-regulated in lung adenocarcinomas. Importantly, these genes are amplified in a subset of lung cancer cell lines as well in primary lung tumors. Of clinical significance is our observation that *PPAT*, *PAICS* and *PKM2* (isoform of the PKM gene) transcript levels correlate with patient survival, differentiation status and smoking status. Furthermore, we also showed that *PAICS* protein expression predicts disease outcome. As indicated earlier, a clear molecular stratification has been instrumental in early detection and precise therapeutic regimen. In addition, we showed the critical role of *PPAT* and *PAICS* in lung cancer progression both *in vitro* and *in vivo*. Our study therefore reveals that *PPAT* and *PAICS* genes as potential therapeutic targets.

The *PKM* gene expresses two isoforms, *PKM1* and *PKM2* both differing by presence of a single exon in a mutually exclusive manner [25, 53]. An earlier study has shown correlation of high *PKM2* levels and poor prognosis and demonstrated *in vitro* and *in vivo* role in lung cancer [54]. We demonstrated that specific knockdown of the PKM2 isoform dramatically reduces cell proliferation in wider spectrum of lung cancer cell lines including lung adenocarcinomas and large cell carcinoma. Recently SAICAR, the intermediate metabolite of the enzyme *PAICS* was shown to allosterically activate PKM2 activity under glucose depleting conditions [24] and mediate ERK1/2 phosphorylation thereby induces cancer cell proliferation [55, 56]. Interestingly, we discovered that *PPAT* and *PAICS* knockdown substantially diminished pyruvate kinase activity that is comparable to the *PKM2* knockdown suggesting that *PKM2* could be an important contributor of the pyruvate kinase activity in lung cancer cell lines. *PKM2* isoform expression which is predominant in cancers, exists as both dimer and tetramer and plays a role in tumorigenesis in both forms [57]. Our study confirms increased *PKM2* expression in lung cancer and regulation of pyruvate activity by *de novo* purine pathway genes can alter metabolic status and promote oncogenesis of lung cells. The oncogenic role of *PPAT* and *PAICS* that we observed therefore could be because of direct contribution to purine supply for efficient DNA replication during S-phase, as observed by our cell-cycle analyses and potentiating pyruvate kinase possibly through PKM2 activity.

*De novo* purine biosynthesis utilizes charged ribose-5-phosphate, known as phosphoribosyl pyrophosphate (PRPP) and glutamine as substrates by *PPAT* in the first committed step. Glutamine, a non-essential amino acid for normal cells, is essential for neoplastic transformation. Moreover most of the cancer cells won't survive in the absence of exogenous glutamine, and show glutamine addiction [58]. Recent study has shown that NSCLC cells show glutamine dependency [59]. Our studies confirmed that glutamine strongly effects lung cancer cell growth as well as in regulating pyruvate kinase activity. We showed that glutamine deprivation reduces *PPAT* and *PAICS* expression. Glutamine has multi-faceted function in cancer metabolism; including as primary carbon source replacing glucose [45, 58]. Substantial evidence suggests that increased uptake of glutamine in cancer cells undergoes the anapleurotic pathway, where it is converted into glutamate and then α-ketoglutarate (α-KG), a key intermediate in the tricarboxylic acid cycle [21]. We also observed that glutamine mediated induction of *PPAT* and *PAICS* expression and PKM2 activity which could be reversed by glutamine antagonist L-diazo-5-oxo-L-norleucine (DON). Glutamine dependency has been demonstrated in multiple cancers [58]. Direct targeting of glutamine dependency by administration of glutamine analogues such as DON, azaserine and acivicin showed their strongest effects on nucleotide biosynthesis [45]. These glutamine analogues showed potent anti-neoplastic effects *in vitro* [48]. However, efforts to incorporate them in clinical settings were stymied because of neurotoxicity, GI toxicity and myelosuppression [45, 58]. A concerted effort was made to circumvent toxicity issues by blocking glutamine availability to cancer cells either through inhibiting its uptake using GPNA(γ-L-glutamyl-p-nitroanilide) in lung cancer [60], or preventing its synthesis by inhibiting glutamine synthase using inhibitors such as BPTES(Bis-2-(5-phenylacetamido-1, 2, 4-thiadiazol-2-yl)ethyl sulfide) [61], compound 968 [62] and L-asparaginase [63]. Knocking down SLC1A5 [Solute Carrier Family 1 (Neutral Amino Acid Transporter), Member 5], the glutamine transporter has been demonstrated to inhibit tumor formation in acute myeloid mouse xenotransplant model [63]. Our study further illustrates the important role played by glutamine in inducing *de novo* purine biosynthetic genes PPAT and PAICS. We suggest that oncogenic effects of glutamine can be mitigated by potentially interfering with *de novo* purine biosynthesis pathway genes such as PPAT and PAICS or PKM2 activity. Inhibiting *de novo* purine pathway and PKM2 activity would also circumvent toxicity issues associated with glutamine antagonists as these genes are overexpressed in cancer cells.

In summary, we identified increased expression of PPAT and PAICS in *de novo* purine biosynthetic pathway that is potentially useful for lung cancer prognosis. Our studies indicated that both genes are amplified in a fraction of lung cancer patients. We demonstrated the functional significance of PPAT and PAICS during oncogenesis through several *in vitro* and *in vivo* studies and their role in regulating PKM2 activity. We established glutamine-mediated induction of PPAT and PAICS as an important metabolic event for increased expression of these genes during lung oncogenesis, which in turn enhances tumor-specific pyruvate kinase activity. Altogether, these results identify potentially novel targets for therapeutic intervention to treat lung cancer.

## MATERIALS AND METHODS

### Patient samples

We used frozen primary tumors and matched non-malignant lung samples from lung adenocarcinoma patients who underwent resection at the University of Michigan Health System from 1991–2007. Informed consent was obtained for each subject and clinical investigations were conducted after approval by the Institutional Review Board. Tumor specimens were immediately frozen following resection and stored at −80°C. Regions containing a minimum of 70% tumor cellularity were used for RNA/protein isolation. Tumor grade assessment as well as histopathological analysis of sections adjacent to regions used for RNA isolation was performed according the IASLC/ATS/ERS International Multidisciplinary Classification of Lung Adenocarcinoma [64, 65]. None of the patients included in this study received preoperative radiation or chemotherapy. Clinical data was retrospectively collected from the medical records and all cases were staged according to the revised 7th TNM classification criteria [65]. The median follow-up time was 8.12 years among the patients that remained alive.

### Primary tumor-derived gene expression data sets

Two published Affymetrix microarray data sets were used in the survival analysis for genes *PPAT*, *PAICS* and *PKM2* [35, 40]. The CEL files of microarray data were normalized using Robust Multi-array Average (RMA) method. The patient characteristics for Shedden et al, 442 tumors are provided in Supplementary Table S1. Overall survival is the outcome for all datasets, and it is censored at 5 years.

### Cell line treatments

The cell lines used in this study were purchased from ATCC, USA. Lung cancer cell lines A549, H23, H661 and H838 were grown in 10% FBS-RPMI 1640 (Life Technologies, CA) in 5% CO_2_ cell culture incubator. The reagents L-glutamine and L-diazo-5-oxo-L-norleucine (DON) are purchased from Sigma-Aldrich, USA. As described elsewhere [60, 66, 67], for glutamine studies, the cells were plated in 6-well tissue culture plates at a density of 0.2 million cells/well. The following day, the cells were washed twice with serum free glutamine-free medium and replaced with 10% dialyzed FBS containing glutamine-free RPMI 1640 (Life Technologies, CA), and then incubated for 48 h. The corresponding glutamine-replete medium was prepared by addition of indicated concentration of glutamine. For DON (a glutamine analogue) studies, we added indicated concentration of DON to the cells which are already in 10% dialyzed FBS + 2 mM Gln+ RPMI 1640 medium [68, 69]. Bronchial lung epithelial cells, BEAS-2B were grown in BEBM medium with supplements (Lonza, USA). Adenoviruses and lentiviruses were generated by the University of Michigan Vector Core. Lung cancer cells were infected with lentiviruses expressing *PPAT* shRNAs, *PAICS* shRNAs or Non-target controls only, and stable cell lines were generated by selection with 1 μg/ml puromycin (Life Technologies, CA).

### Pyruvate kinase activity assay

The pyruvate kinase activity was measured using Pyruvate Kinase Assay kit (Biovision, Catalog #K709-100). Briefly, cell pellets were suspended in 4x assay buffer (10,000 cells/100 μl). The lysed cells were spun (10,000 rpm/5 mins) to clear cell debris and cell supernatant was used to measure pyruvate kinase activity colorimetrically (λ = 570 nm) using 96 well-plate assays. The PK activity as measured by pyruvate production was calculated using standard PK activity provided by the kit. The calculated activity was then normalized to protein concentration of the cell lysate and plotted. The y-axis represents the percentage normalized PK activity (% μmol pyruvate produced/mg of protein).

### Immunoblot analysis

For immunoblot analysis, 10 μg of lung adenocarcinoma tissue lysates from regions >70% tumor cellularity and matched normal tissue lysates were used. Tissue and lung cancer cell line lysates were boiled in sample buffer, separated by sodium dodecyl sulfate-polyacrylamide gel electrophoresis, and transferred onto polyvinylidene difluoride membrane (GE Healthcare, Piscataway, NJ). First lane contains 5 ul of spectra multicolor broad range protein ladder (Life Technologies, CA). The membrane was incubated for 1 hour in blocking buffer (TBS, 0.05% Tween, 5% nonfat dry milk) and incubated overnight at 4°C with respective primary antibodies, and signals were visualized after incubating with secondary antibody conjugated with HRP. The list of various antibodies, catalogue numbers and dilutions used are mentioned in Supplementary Table S2.

### RNA and DNA isolation and quantitative real-time PCR assays

Total RNA was isolated from tissues and cells using RNeasy Mini Kit (Qiagen, USA). Quantitative PCR (qRT-PCR) with cDNA (Applied Biosystems, USA) was performed with 10 ng of cDNA used as template and quantified using SYBR (Applied Biosystems, USA). qRT-PCR assays were performed in triplicate. Both PKM1 and PKM2 qRT-PCR primers were taken from elsewhere [43]. The primer sequences for the transcript analyzed by SYBR green qRT-PCR as provided in Supplementary Table S3.

### Immunohistochemistry of TMA

Immunohistochemistry on lung adenocarcinoma tissue microarray was performed using PPAT mouse monoclonal antibody, PAICS mouse monoclonal antibody and PKM2 specific rabbit monoclonal antibody. Immunohistochemistry was performed using an automated protocol developed for the DISCOVERY XT automated slide staining system (Ventana Medical Systems, Inc.,) using Ultramap anti-mouse HRP or Ultramap anti-rabbit HRP as secondary antibody and was detected using ChromoMap DAB (Cat#760-159, Ventana Medical Systems Inc.,) Hematoxylin II (Cat#790-2208 Ventana-Roche, Tucson, AZ, USA) was used as the counterstain. Immunohistochemistry staining was evaluated by D.T. TMA sections were scored for the proportion of tumor cells staining for the marker and intensity using the methodology described by Harvey et al [70].

### Fluorescence *in situ* hybridization (FISH)

BAC clones were used to generate the locus specific (4q12) FISH probe for PAICS and PPAT (RP11-393M11) and chromosome 4 control probe (RP11-719O22). All clones were tested on normal human metaphase chromosomes to validate map position. PAICS locus probe and chromosome 4 control probes were detected with anti-digoxigenin fluorescein Fab fragments to yield green color and Streptavidin Alexa fluor 594 to yield red color, respectively. 200 ml overnight cultures for each BAC clone were grown in LB medium containing 12.5 μg/ml of chloramphenicol at 37°C for 14–16 hours with constant shaking. DNA was prepared using Qiagen-midiprep kit using Qiatip-100 according to the protocol provided by the manufacturer (Qiagen, USA).

All FISH probes were prepared by nick translation labeling using modified nucleotides conjugated with biotin or digoxigenin utilizing biotin nick translation mix (Cat# 11745824910, Roche, USA) for chromosome 4 control probe; digoxigenin nick translation mix (Cat# 11745816910, Roche, USA) for PAICS locus probe, respectively. Probe DNA was precipitated and dissolved in hybridization mixture containing 50% formamide, 2XSSC, 10% dextran sulphate, and 1% Denhardt's solution. Approximately 200ng of each labeled probe was used for hybridization. Fluorescent signals were detected with Streptavidin Alexa fluor 594 (Cat# S-32356, Invitrogen, USA) and anti-digoxigenin fluorescein Fab fragments (Cat# 11207741910, Roche, USA) for red and green colors, respectively. FISH scoring was performed by an experienced cytogeneticist (NP). Copy number was evaluated based on the ratio between control and locus specific probe. Copy number 4 or above are considered as amplification in at least 15–20% of the cells in the tumor samples. Fluorescent images were captured using a high resolution CCD camera controlled by ISIS image processing software (Metasystems, Germany).

### siRNA transfections

Custom made small interfering RNA (siRNA) duplexes for RNA interference of *PPAT*, *PAICS* were purchased from Dharmacon, Lafayette, CO (GE Healthcare, USA). PKM2 specific siRNA was custom synthesized from Dharmacon, Lafayette, CO (GE Healthcare, USA) [43]. The list of siRNAs, shRNAs and the catalogue numbers are mentioned in Supplementary Table S4. Transfections were performed with either oligofectamine (Life Technologies, USA) or Lipofectamine^®^ RNAimax (Life technologies, USA). Seventy-two hours after the siRNA transfections, the cells were harvested for RNA isolation or lysed for immunoblot analysis. Short hairpin RNA (shRNA) constructs were generated using pGreen-puro vector (System Biosciences, Mountain View, CA). For PPAT knockdown, the pGIPz plasmids were obtained from Dharmacon, Lafayette, CO (GE Healthcare, USA). All the lentiviruses were generated by the University of Michigan Vector Core.

### Cell proliferation assays

Cell proliferation was measured by cell counting. For this, stable and/or transient PAICS, PPAT and PKM2 knockdowns were used. Around 10,000 cells/well were seeded in 24-well plates (*n* = 4), after transfecting either with specific siRNA duplex or stable knockdowns as described above. Stable knockdown of PAICS was performed using shRNA strategy using lentiviral construct with specific duplex sequences targeting PAICS. These stable knock down cells were plated at same density as mentioned earlier, harvested and counted at specified time points by Coulter counter (Beckman Coulter, Fullerton, CA). Non-targeting siRNA or shRNA-treated cells were served as controls. For glutamine studies, as mentioned earlier lung cancer cell lines were seeded in same density in glutamine-free medium and counted the cells after each indicated time point. Each experiment has been carried at thrice with four replicates in each experiment per sample. Data shown is representative figure of one such experiment.

### Cell cycle analysis

siRNA treated cells (as mentioned earlier) were trypsinized and resuspended in 0.5 ml of PBS at a concentration of 10^6^cells/ml. Ice-cold absolute ethanol was added in equal volumes to the sample. The sample was incubated for minimum of 20 min, followed by spin at 2000 rpm for 2–5 min. Ethanol was decanted and cell pellet was resuspended in 0.5 ml propidium iodide+RNase solution and incubated at room temperature for minimum of 20 min in dark. The cell cycle analysis was carried through flow cytometry core facility, University of Michigan, Ann Arbor. Each experiment has been carried at twice in triplicate per sample. Data shown is representative figure of one such experiment.

### Matrigel invasion assay

For invasion assays, transiently transfected or stable knockdown of PPAT, PAICS and PKM2 in were used in A549 cells. BEAS-2B (benign transformed lung epithelial) cells were infected with PAICS-adenovirus. Seventy-two hours post-transfection and/or infection, cells were seeded onto BD BioCoat matrigel matrix (BD, CA) present in the insert of a 24-well culture plate. For A549, 10% serum containing RPMI medium was added to the lower chamber as a chemoattractant. For BEAS-2B, BEBM medium with supplementary growth factors was added as chemoattractant. After 36 h, the non-invading cells and matrigel matrix were gently removed with a cotton swab. Invasive cells located on the lower side of the chamber were stained with 0.2% crystal violet in methanol, air-dried and photographed. They were then enumerated microscopically using multiple representative areas. For colorimetric assays, the inserts were treated with 150 μl of 10% acetic acid and the absorbance measured at 560 nm [71]. Each experiment has been carried twice in triplicates per sample. Data shown is representative figure of one such experiment.

### *In vitro* overexpression

PAICS cDNA (Origene, MD; Cat# RC223925) was cloned into Gateway expression system (Life Technologies CA). To generate adenoviral construct, PCR8-PAICS (flag tagged) was recombined with pAD/CMV/V5-Dest™ (Life Technologies, CA) respectively using LR Clonase II (Life Technologies, CA) [71]. Adenoviruses were generated by the University of Michigan Vector Core. Benign lung epithelial cells (BEAS2-B) were infected with adenoviruses expressing PA1CS or lacZ for transient over-expression.

### Chicken chorioallantoic membrane (CAM) assay

The CAM assay for tumor (or xenograft) formation was performed as previously described [49, 51, 72]. Briefly, fertilized eggs were incubated in a rotary humidified incubator at 38°C for 10 days. CAM was dropped by making two holes, one through the eggshell into the air sac and a second hole near the allantoic vein that penetrates the eggshell membrane but not the CAM. Subsequently, Dremel 3000 Rotary Tool was used to cut a 1 cm^2^ window to expose the underlying CAM near the allantoic vein. Approximately 2 million cells in 50 μl of media were implanted in each egg, windows were sealed and the eggs were returned to the incubator. Following 7-day incubation post-tumor cells injection; the primary tumor was excised and weighed. Around 8 eggs per group were used in each experiment.

### Tumor xenograft model

All procedures involving mice were approved by the University Committee on Use and Care of Animals at the University of Michigan and conform to their relevant regulatory standards. To evaluate the role of PPAT and PAICS in tumor formation, we propagated stable PPAT and PAICS knockdown A549 pools using two-independent shRNAs, and Non-targeting shRNA control cells and inoculated 1 × 10^6^ cells subcutaneously into the dorsal flank of 4-week-old male athymic nude mice (*Foxn1^nu^*) (*n* = 8 for each group; Harlan Laboratories, USA). Tumor size was measured biweekly, and tumor volumes were calculated using the formula (π/6) (L × W^2^), where L = length of tumor and W = width and represented as mm^3^/tumor. Post-monitoring, tumors were dissected out, photographed and weighed. Tumor weight is represented in mg/tumor.

### Statistical evaluation

For cell lines and pyruvate kinase assays representative readings in triplicate was averaged and plotted in various graphs. The error indicates the standard deviation (SD) in case of colorimetric assays and cell counts. In case of tumor grafts, the error bars represent standard error (SE) instead of SD. For CAM assays, 8 eggs have been used per treatment while for mice experiments, values represent average of 8 mice/group. Statistical evaluation of the data was carried out using student's *t*-test and values that showed *P* <0.05 were considered significant.

The clinical and pathological characteristics were analyzed using the Student's *t*-test for comparing two groups and by ANOVA for multiple group comparisons, with *P* < 0.05 considered statistically significant. For values that were not normally distributed the Mann-Whitney rank sum test was used. Univariate and multivariate (adjusted by age, gender, stage and differentiation) Cox proportional hazards regression model were used for continuous values of each gene; Kaplan-Meier survival curve and log-rank test were used to separate patients into three risk tertiles (high, medium, and low-risk, 1/3rd in each group) based on gene/protein expression levels.

## SUPPLEMENTARY DATA, FIGURES AND TABLES



## References

[1] Siegel RL, Miller KD, Jemal A (2015). Cancer statistics, 2015. CA Cancer J Clin.

[2] Noguchi M, Morikawa A, Kawasaki M, Matsuno Y, Yamada T, Hirohashi S, Kondo H, Shimosato Y (1995). Small adenocarcinoma of the lung. Histologic characteristics and prognosis. Cancer.

[3] Gabrielson E (2006). Worldwide trends in lung cancer pathology. Respirology.

[4] Pao W, Chmielecki J (2010). Rational, biologically based treatment of EGFR-mutant non-small-cell lung cancer. Nature reviews Cancer.

[5] Pao W, Hutchinson KE (2012). Chipping away at the lung cancer genome. Nature medicine.

[6] Levy MA, Lovly CM, Pao W (2012). Translating genomic information into clinical medicine: lung cancer as a paradigm. Genome research.

[7] Ou SH, Bazhenova L, Camidge DR, Solomon BJ, Herman J, Kain T, Bang YJ, Kwak EL, Shaw AT, Salgia R, Maki RG, Clark JW, Wilner KD, Iafrate AJ (2010). Rapid and dramatic radiographic and clinical response to an ALK inhibitor (crizotinib, PF02341066) in an ALK translocation-positive patient with non-small cell lung cancer. Journal of thoracic oncology: official publication of the International Association for the Study of Lung Cancer.

[8] Kwak EL, Bang YJ, Camidge DR, Shaw AT, Solomon B, Maki RG, Ou SH, Dezube BJ, Janne PA, Costa DB, Varella-Garcia M, Kim WH, Lynch TJ, Fidias P, Stubbs H, Engelman JA (2010). Anaplastic lymphoma kinase inhibition in non-small-cell lung cancer. The New England journal of medicine.

[9] Fernandez-Cuesta L, Plenker D, Osada H, Sun R, Menon R, Leenders F, Ortiz-Cuaran S, Peifer M, Bos M, Dassler J, Malchers F, Schottle J, Vogel W, Dahmen I, Koker M, Ullrich RT (2014). CD74-NRG1 Fusions in Lung Adenocarcinoma. Cancer discovery.

[10] Nakaoku T, Tsuta K, Ichikawa H, Shiraishi K, Sakamoto H, Enari M, Furuta K, Shimada Y, Ogiwara H, Watanabe S, Nokihara H, Yasuda K, Hiramoto M, Nammo T, Ishigame T, Schetter AJ (2014). Druggable oncogene fusions in invasive mucinous lung adenocarcinoma. Clinical cancer research: an official journal of the American Association for Cancer Research.

[11] Jin G, Reitman ZJ, Duncan CG, Spasojevic I, Gooden DM, Rasheed BA, Yang R, Lopez GY, He Y, McLendon RE, Bigner DD, Yan H (2013). Disruption of wild-type IDH1 suppresses D-2-hydroxyglutarate production in IDH1-mutated gliomas. Cancer research.

[12] Reitman ZJ, Parsons DW, Yan H (2010). IDH1 and IDH2: not your typical oncogenes. Cancer cell.

[13] Sreekumar A, Poisson LM, Rajendiran TM, Khan AP, Cao Q, Yu J, Laxman B, Mehra R, Lonigro RJ, Li Y, Nyati MK, Ahsan A, Kalyana-Sundaram S, Han B, Cao X, Byun J (2009). Metabolomic profiles delineate potential role for sarcosine in prostate cancer progression. Nature.

[14] Khan AP, Rajendiran TM, Ateeq B, Asangani IA, Athanikar JN, Yocum AK, Mehra R, Siddiqui J, Palapattu G, Wei JT, Michailidis G, Sreekumar A, Chinnaiyan AM (2013). The role of sarcosine metabolism in prostate cancer progression. Neoplasia.

[15] Ying H, Kimmelman AC, Lyssiotis CA, Hua S, Chu GC, Fletcher-Sananikone E, Locasale JW, Son J, Zhang H, Coloff JL, Yan H, Wang W, Chen S, Viale A, Zheng H, Paik JH (2012). Oncogenic Kras maintains pancreatic tumors through regulation of anabolic glucose metabolism. Cell.

[16] Liu YC, Li F, Handler J, Huang CR, Xiang Y, Neretti N, Sedivy JM, Zeller KI, Dang CV (2008). Global regulation of nucleotide biosynthetic genes by c-Myc. PloS one.

[17] Barfeld SJ, Fazli L, Persson M, Marjavaara L, Urbanucci A, Kaukoniemi KM, Rennie PS, Ceder Y, Chabes A, Visakorpi T, Mills IG (2015). Myc-dependent purine biosynthesis affects nucleolar stress and therapy response in prostate cancer. Oncotarget.

[18] Eissmann M, Schwamb B, Melzer IM, Moser J, Siele D, Kohl U, Rieker RJ, Wachter DL, Agaimy A, Herpel E, Baumgarten P, Mittelbronn M, Rakel S, Kogel D, Bohm S, Gutschner T (2013). A functional yeast survival screen of tumor-derived cDNA libraries designed to identify anti-apoptotic mammalian oncogenes. PloS one.

[19] Warburg O (1956). On respiratory impairment in cancer cells. Science.

[20] Vander Heiden MG, Cantley LC, Thompson CB (2009). Understanding the Warburg effect: the metabolic requirements of cell proliferation. Science.

[21] Vander Heiden MG, Lunt SY, Dayton TL, Fiske BP, Israelsen WJ, Mattaini KR, Vokes NI, Stephanopoulos G, Cantley LC, Metallo CM, Locasale JW (2011). Metabolic pathway alterations that support cell proliferation. Cold Spring Harbor symposia on quantitative biology.

[22] Hanahan D, Weinberg RA (2011). Hallmarks of cancer: the next generation. Cell.

[23] Cory JG, Cory AH (2006). Critical roles of glutamine as nitrogen donors in purine and pyrimidine nucleotide synthesis: asparaginase treatment in childhood acute lymphoblastic leukemia. *In vivo*.

[24] Keller KE, Tan IS, Lee YS (2012). SAICAR stimulates pyruvate kinase isoform M2 and promotes cancer cell survival in glucose-limited conditions. Science.

[25] Tamada M, Suematsu M, Saya H (2012). Pyruvate kinase M2: multiple faces for conferring benefits on cancer cells. Clinical cancer research : an official journal of the American Association for Cancer Research.

[26] Yang W, Xia Y, Hawke D, Li X, Liang J, Xing D, Aldape K, Hunter T, Alfred Yung WK, Lu Z (2012). PKM2 phosphorylates histone H3 and promotes gene transcription and tumorigenesis. Cell.

[27] Garber ME, Troyanskaya OG, Schluens K, Petersen S, Thaesler Z, Pacyna-Gengelbach M, van de Rijn M, Rosen GD, Perou CM, Whyte RI, Altman RB, Brown PO, Botstein D, Petersen I (2001). Diversity of gene expression in adenocarcinoma of the lung. Proc Natl Acad Sci U S A.

[28] Beer DG, Kardia SL, Huang CC, Giordano TJ, Levin AM, Misek DE, Lin L, Chen G, Gharib TG, Thomas DG, Lizyness ML, Kuick R, Hayasaka S, Taylor JM, Iannettoni MD, Orringer MB (2002). Gene-expression profiles predict survival of patients with lung adenocarcinoma. Nature medicine.

[29] Talbot SG, Estilo C, Maghami E, Sarkaria IS, Pham DK, P Oc, Socci ND, Ngai I, Carlson D, Ghossein R, Viale A, Park BJ, Rusch VW, Singh B (2005). Gene expression profiling allows distinction between primary and metastatic squamous cell carcinomas in the lung. Cancer research.

[30] Landi MT, Dracheva T, Rotunno M, Figueroa JD, Liu H, Dasgupta A, Mann FE, Fukuoka J, Hames M, Bergen AW, Murphy SE, Yang P, Pesatori AC, Consonni D, Bertazzi PA, Wacholder S (2008). Gene expression signature of cigarette smoking and its role in lung adenocarcinoma development and survival. PloS one.

[31] Hou J, Aerts J, den Hamer B, van Ijcken W, den Bakker M, Riegman P, van der Leest C, van der Spek P, Foekens JA, Hoogsteden HC, Grosveld F, Philipsen S (2010). Gene expression-based classification of non-small cell lung carcinomas and survival prediction. PloS one.

[32] Okayama H, Kohno T, Ishii Y, Shimada Y, Shiraishi K, Iwakawa R, Furuta K, Tsuta K, Shibata T, Yamamoto S, Watanabe S, Sakamoto H, Kumamoto K, Takenoshita S, Gotoh N, Mizuno H (2012). Identification of genes upregulated in ALK-positive and EGFR/KRAS/ALK-negative lung adenocarcinomas. Cancer research.

[33] Selamat SA, Chung BS, Girard L, Zhang W, Zhang Y, Campan M, Siegmund KD, Koss MN, Hagen JA, Lam WL, Lam S, Gazdar AF, Laird-Offringa IA (2012). Genome-scale analysis of DNA methylation in lung adenocarcinoma and integration with mRNA expression. Genome research.

[34] Rhodes DR, Kalyana-Sundaram S, Mahavisno V, Varambally R, Yu J, Briggs BB, Barrette TR, Anstet MJ, Kincead-Beal C, Kulkarni P, Varambally S, Ghosh D, Chinnaiyan AM (2007). Oncomine 3.0: genes, pathways, and networks in a collection of 18, 000 cancer gene expression profiles. Neoplasia.

[35] Shedden K, Taylor JM, Enkemann SA, Tsao MS, Yeatman TJ, Gerald WL, Eschrich S, Jurisica I, Giordano TJ, Misek DE, Chang AC, Zhu CQ, Strumpf D, Hanash S, Shepherd FA, Ding K (2008). Gene expression-based survival prediction in lung adenocarcinoma: a multi-site, blinded validation study. Nature medicine.

[36] Seo JS, Ju YS, Lee WC, Shin JY, Lee JK, Bleazard T, Lee J, Jung YJ, Kim JO, Shin JY, Yu SB, Kim J, Lee ER, Kang CH, Park IK, Rhee H (2012). The transcriptional landscape and mutational profile of lung adenocarcinoma. Genome research.

[37] Cerami E, Gao J, Dogrusoz U, Gross BE, Sumer SO, Aksoy BA, Jacobsen A, Byrne CJ, Heuer ML, Larsson E, Antipin Y, Reva B, Goldberg AP, Sander C, Schultz N (2012). The cBio cancer genomics portal: an open platform for exploring multidimensional cancer genomics data. Cancer discovery.

[38] Gao J, Aksoy BA, Dogrusoz U, Dresdner G, Gross B, Sumer SO, Sun Y, Jacobsen A, Sinha R, Larsson E, Cerami E, Sander C, Schultz N (2013). Integrative analysis of complex cancer genomics and clinical profiles using the cBioPortal. Science signaling.

[39] Dhanasekaran SM, Alejandro Balbin O, Chen G, Nadal E, Kalyana-Sundaram S, Pan J, Veeneman B, Cao X, Malik R, Vats P, Wang R, Huang S, Zhong J, Jing X, Iyer M, Wu YM (2014). Transcriptome meta-analysis of lung cancer reveals recurrent aberrations in NRG1 and Hippo pathway genes. Nat Commun.

[40] Bild AH, Yao G, Chang JT, Wang Q, Potti A, Chasse D, Joshi MB, Harpole D, Lancaster JM, Berchuck A, Olson JA, Marks JR, Dressman HK, West M, Nevins JR (2006). Oncogenic pathway signatures in human cancers as a guide to targeted therapies. Nature.

[41] Ramos AH, Dutt A, Mermel C, Perner S, Cho J, Lafargue CJ, Johnson LA, Stiedl AC, Tanaka KE, Bass AJ, Barretina J, Weir BA, Beroukhim R, Thomas RK, Minna JD, Chirieac LR (2009). Amplification of chromosomal segment 4q12 in non-small cell lung cancer. Cancer biology & therapy.

[42] Lunt SY, Vander Heiden MG (2011). Aerobic glycolysis: meeting the metabolic requirements of cell proliferation. Annual review of cell and developmental biology.

[43] Goldberg MS, Sharp PA (2012). Pyruvate kinase M2-specific siRNA induces apoptosis and tumor regression. The Journal of experimental medicine.

[44] Bronder JL, Moran RG (2002). Antifolates targeting purine synthesis allow entry of tumor cells into S phase regardless of p53 function. Cancer research.

[45] Hensley CT, Wasti AT, DeBerardinis RJ (2013). Glutamine and cancer: cell biology, physiology, and clinical opportunities. The Journal of clinical investigation.

[46] Kvamme E, Svenneby G (1960). Effect of anaerobiosis and addition of keto acids on glutamine utilization by Ehrlich ascites-tumor cells. Biochimica et biophysica acta.

[47] Drogat B, Bouchecareilh M, North S, Petibois C, Deleris G, Chevet E, Bikfalvi A, Moenner M (2007). Acute L-glutamine deprivation compromises VEGF-a upregulation in A549/8 human carcinoma cells. Journal of cellular physiology.

[48] Ahluwalia GS, Grem JL, Hao Z, Cooney DA (1990). Metabolism and action of amino acid analog anti-cancer agents. Pharmacology & therapeutics.

[49] Brenner JC, Ateeq B, Li Y, Yocum AK, Cao Q, Asangani IA, Patel S, Wang X, Liang H, Yu J, Palanisamy N, Siddiqui J, Yan W, Cao X, Mehra R, Sabolch A (2011). Mechanistic rationale for inhibition of poly(ADP-ribose) polymerase in ETS gene fusion-positive prostate cancer. Cancer cell.

[50] Prensner JR, Iyer MK, Sahu A, Asangani IA, Cao Q, Patel L, Vergara IA, Davicioni E, Erho N, Ghadessi M, Jenkins RB, Triche TJ, Malik R, Bedenis R, McGregor N, Ma T (2013). The long noncoding RNA SChLAP1 promotes aggressive prostate cancer and antagonizes the SWI/SNF complex. Nature genetics.

[51] Leupold JH, Yang HS, Colburn NH, Asangani I, Post S, Allgayer H (2007). Tumor suppressor Pdcd4 inhibits invasion/intravasation and regulates urokinase receptor (u-PAR) gene expression via Sp-transcription factors. Oncogene.

[52] Stewart B, Wild C.P (2014). World Cancer Report.

[53] Noguchi T, Yamada K, Inoue H, Matsuda T, Tanaka T (1987). The L- and R-type isozymes of rat pyruvate kinase are produced from a single gene by use of different promoters. The Journal of biological chemistry.

[54] Peng XC, Gong FM, Zhao YW, Zhou LX, Xie YW, Liao HL, Lin HJ, Li ZY, Tang MH, Tong AP (2011). Comparative proteomic approach identifies PKM2 and cofilin-1 as potential diagnostic, prognostic and therapeutic targets for pulmonary adenocarcinoma. PloS one.

[55] Keller KE, Doctor ZM, Dwyer ZW, Lee YS (2014). SAICAR induces protein kinase activity of PKM2 that is necessary for sustained proliferative signaling of cancer cells. Molecular cell.

[56] SAICAR binding activates PKM2 protein kinase activity (2014). Cancer discovery.

[57] Gao X, Wang H, Yang JJ, Liu X, Liu ZR (2012). Pyruvate kinase M2 regulates gene transcription by acting as a protein kinase. Molecular cell.

[58] Wise DR, Thompson CB (2010). Glutamine addiction: a new therapeutic target in cancer. Trends in biochemical sciences.

[59] van den Heuvel AP, Jing J, Wooster RF, Bachman KE (2012). Analysis of glutamine dependency in non-small cell lung cancer: GLS1 splice variant GAC is essential for cancer cell growth. Cancer biology & therapy.

[60] Hassanein M, Hoeksema MD, Shiota M, Qian J, Harris BK, Chen H, Clark JE, Alborn WE, Eisenberg R, Massion PP (2013). SLC1A5 mediates glutamine transport required for lung cancer cell growth and survival. Clinical cancer research: an official journal of the American Association for Cancer Research.

[61] Robinson MM, McBryant SJ, Tsukamoto T, Rojas C, Ferraris DV, Hamilton SK, Hansen JC, Curthoys NP (2007). Novel mechanism of inhibition of rat kidney-type glutaminase by bis-2-(5-phenylacetamido-1, 2, 4-thiadiazol-2-yl)ethyl sulfide (BPTES). The Biochemical journal.

[62] Wang JB, Erickson JW, Fuji R, Ramachandran S, Gao P, Dinavahi R, Wilson KF, Ambrosio AL, Dias SM, Dang CV, Cerione RA (2010). Targeting mitochondrial glutaminase activity inhibits oncogenic transformation. Cancer cell.

[63] Willems L, Jacque N, Jacquel A, Neveux N, Maciel TT, Lambert M, Schmitt A, Poulain L, Green AS, Uzunov M, Kosmider O, Radford-Weiss I, Moura IC, Auberger P, Ifrah N, Bardet V (2013). Inhibiting glutamine uptake represents an attractive new strategy for treating acute myeloid leukemia. Blood.

[64] Van Schil PE, Sihoe AD, Travis WD (2013). Pathologic classification of adenocarcinoma of lung. Journal of surgical oncology.

[65] Travis WD, Rekhtman N (2011). Pathological diagnosis and classification of lung cancer in small biopsies and cytology: strategic management of tissue for molecular testing. Seminars in respiratory and critical care medicine.

[66] Jewell JL, Kim YC, Russell RC, Yu FX, Park HW, Plouffe SW, Tagliabracci VS, Guan KL (2015). Metabolism. Differential regulation of mTORC1 by leucine and glutamine. Science.

[67] Shanware NP, Bray K, Eng CH, Wang F, Follettie M, Myers J, Fantin VR, Abraham RT (2014). Glutamine deprivation stimulates mTOR-JNK-dependent chemokine secretion. Nat Commun.

[68] Shelton LM, Huysentruyt LC, Seyfried TN (2010). Glutamine targeting inhibits systemic metastasis in the VM-M3 murine tumor model. International journal of cancer Journal international du cancer.

[69] Timmerman LA, Holton T, Yuneva M, Louie RJ, Padro M, Daemen A, Hu M, Chan DA, Ethier SP, van 't Veer LJ, Polyak K, McCormick F, Gray JW (2013). Glutamine sensitivity analysis identifies the xCT antiporter as a common triple-negative breast tumor therapeutic target. Cancer cell.

[70] Harvey JM, Clark GM, Osborne CK, Allred DC (1999). Estrogen receptor status by immunohistochemistry is superior to the ligand-binding assay for predicting response to adjuvant endocrine therapy in breast cancer. Journal of clinical oncology: official journal of the American Society of Clinical Oncology.

[71] Chakravarthi BV, Pathi SS, Goswami MT, Cieslik M, Zheng H, Nallasivam S, Arekapudi SR, Jing X, Siddiqui J, Athanikar J, Carskadon SL, Lonigro RJ, Kunju LP, Chinnaiyan AM, Palanisamy N, Varambally S (2014). The miR-124-prolyl hydroxylase P4HA1-MMP1 axis plays a critical role in prostate cancer progression. Oncotarget.

[72] Asangani IA, Rasheed SA, Nikolova DA, Leupold JH, Colburn NH, Post S, Allgayer H (2008). MicroRNA-21 (miR-21) post-transcriptionally downregulates tumor suppressor Pdcd4 and stimulates invasion, intravasation and metastasis in colorectal cancer. Oncogene.

[73] Stearman RS, Dwyer-Nield L, Zerbe L, Blaine SA, Chan Z, Bunn PA, Johnson GL, Hirsch FR, Merrick DT, Franklin WA, Baron AE, Keith RL, Nemenoff RA, Malkinson AM, Geraci MW (2005). Analysis of orthologous gene expression between human pulmonary adenocarcinoma and a carcinogen-induced murine model. The American journal of pathology.

[74] Su LJ, Chang CW, Wu YC, Chen KC, Lin CJ, Liang SC, Lin CH, Whang-Peng J, Hsu SL, Chen CH, Huang CY (2007). Selection of DDX5 as a novel internal control for Q-RT-PCR from microarray data using a block bootstrap re-sampling scheme. BMC genomics.

[75] Wei DC, Yeh YC, Hung JJ, Chou TY, Wu YC, Lu PJ, Cheng HC, Hsu YL, Kuo YL, Chen KY, Lai JM (2012). Overexpression of T-LAK cell-originated protein kinase predicts poor prognosis in patients with stage I lung adenocarcinoma. Cancer science.

