# Supplementary material for: Role and regulation of coordinately expressed de novo purine biosynthetic enzymes PPAT and PAICS in lung cancer
Source: Oncotarget. 2015 Jun 23;6(27):23445–61. doi: 10.18632/oncotarget.4352 (PMC4695129; doi:10.18632/oncotarget.4352)
Supplement: Supplementary file 1 [file oncotarget-06-23445-s001.pdf]

SUPPLEMENTARY DATA

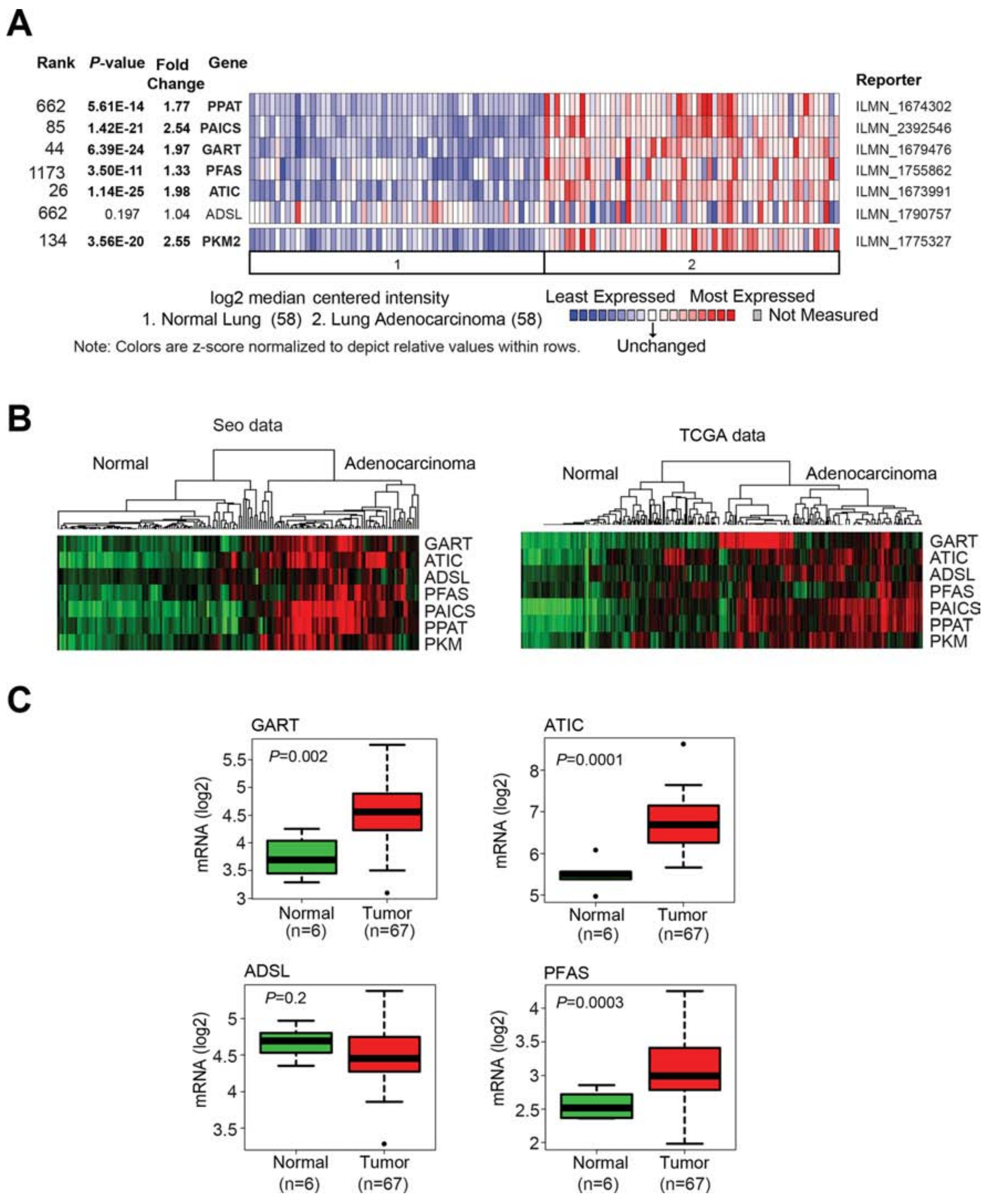

Supplementary Figure S1: *De novo* purine biosynthetic enzymes and pyruvate kinase M expression in lung adenocarcinoma. A. Gene expression profiling studies by Oncomine [1] and **B.** Seo et al(85 AD vs 77 normal) [2] and TCGA(309 AD vs 73 normal) [3, 4] datasets suggest enhanced expression of multiple *de novo* purine biosynthetic enzymes and pyruvate kinase M in lung adenocarcinoma tissues. Expression is represented by a color scale highlighting down-regulation (blue), no alteration (white), and up-regulation (red) of transcripts in S1A. In Figure S1B, down-regulation is represented in green while up-regulation is represented in red. **C.** Quantitative measurement of *GART*, *ATIC, ADSL* and *PFAS* by next-generation RNA sequencing [RPKM (log2)] in lung adenocarcinomas [5]. Related to Figure 1.

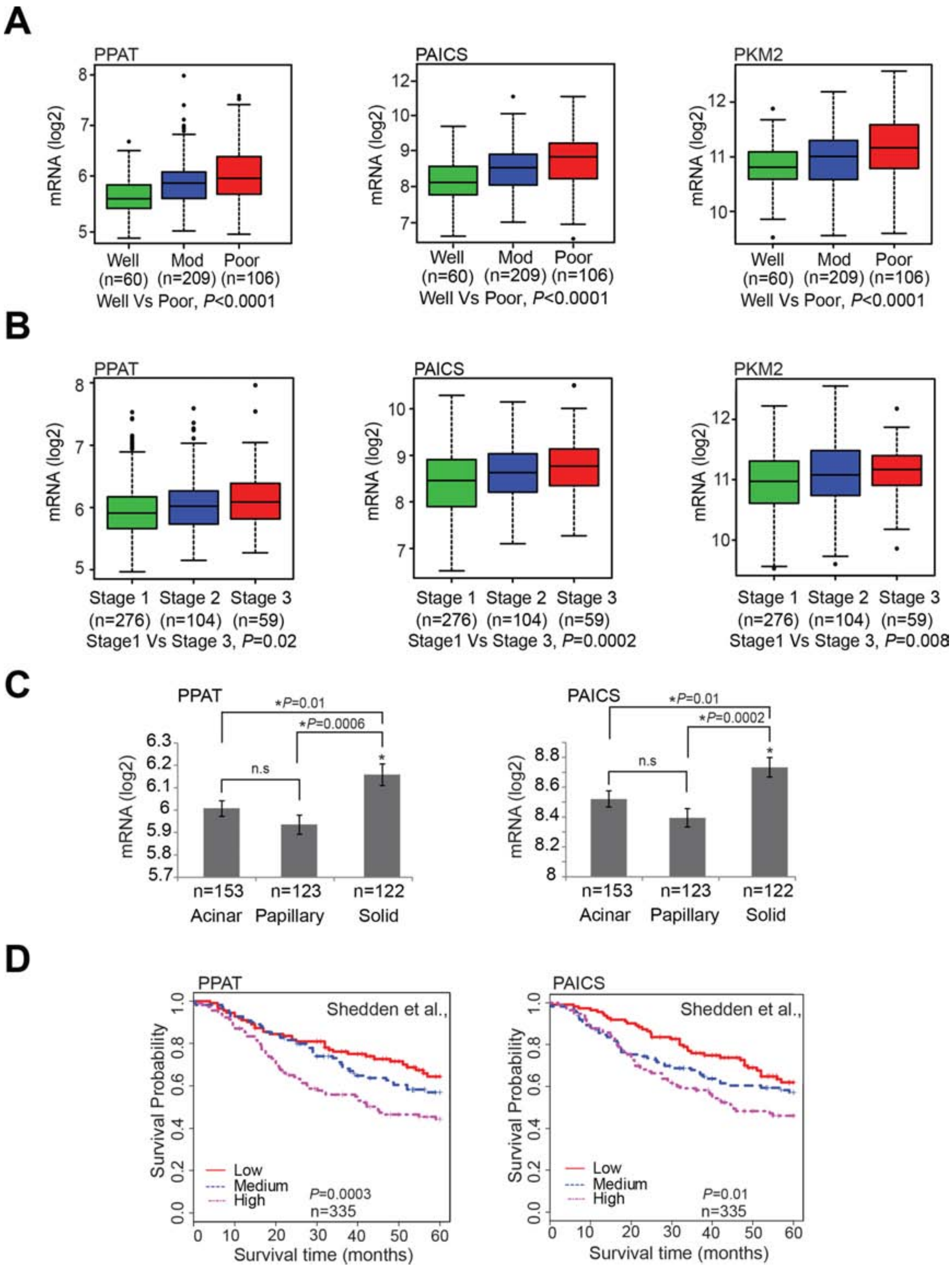

Supplementary Figure S2: Increased expression of *de novo* purine biosynthesis pathway genes, *PPAT* and *PAICS*, and *PKM2* correlates with poor differentiation status and advanced stage of lung cancer. A. Transcript levels of *PPAT*, *PAICS* and *PKM2* based on differentiation levels (well, moderate or poorly differentiated) and **B.** stage specific manner in lung cancers [6]. **C.** *PPAT* and *PAICS* transcript expression based on morphological type of invasive adenocarcinoma (acinar, papillary or solid). **D.** Kaplan–Meier (K-M) analysis of disease free survival (DFS) time according to the *PPAT* and *PAICS* transcript levels as measured using Affymetrix oligonucleotide microarray datasets (data retrieved from $n = 335$ out of 442 adenocarcinomas which have disease free survival information) by Shedden *et al* [6]. Related to Figures 1 and 2.

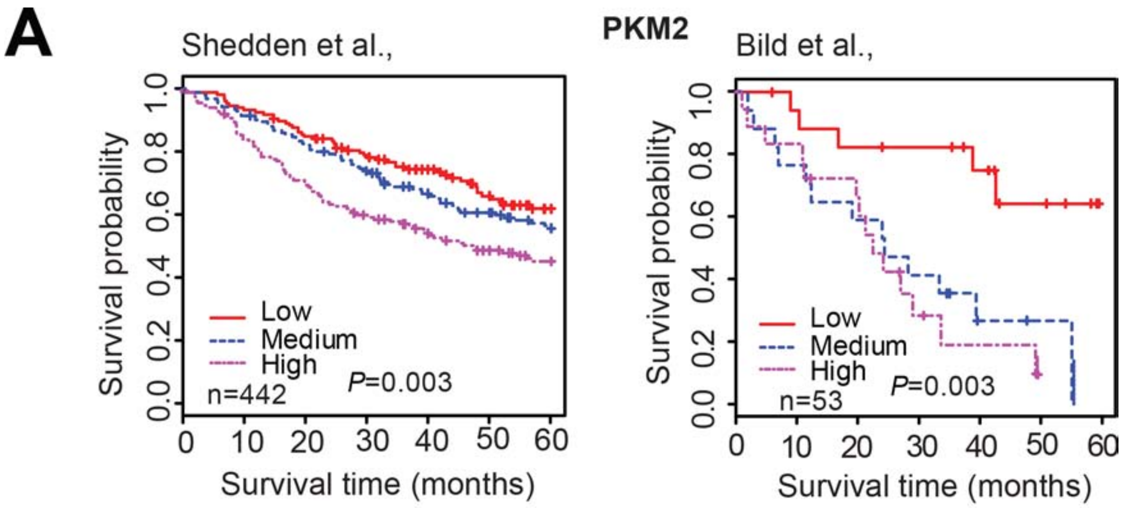

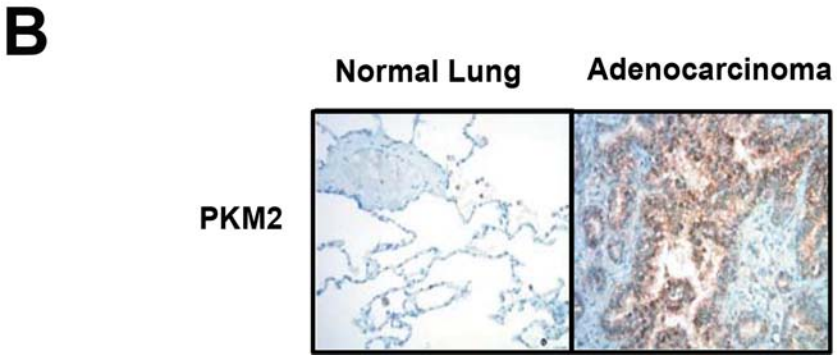

Supplementary Figure S3: Over-expressed *PKM2* correlates with poor prognosis of lung cancer patients and immunohistochemical analyses show increased *PKM2* expression in lung adenocarcinomas. A. Kaplan-Meier analysis of survival time according to *PKM2* transcript levels as measured using affymetrix oligonucleotide datasets by Shedden *et al* [6] and Bild *et al* [7]. **B.** Photomicrographs of *PKM2* immunostaining in Normal Lung (Left) and Adenocarcinoma (Right) using *PKM2* specific antibodies. Related to Figure 2.

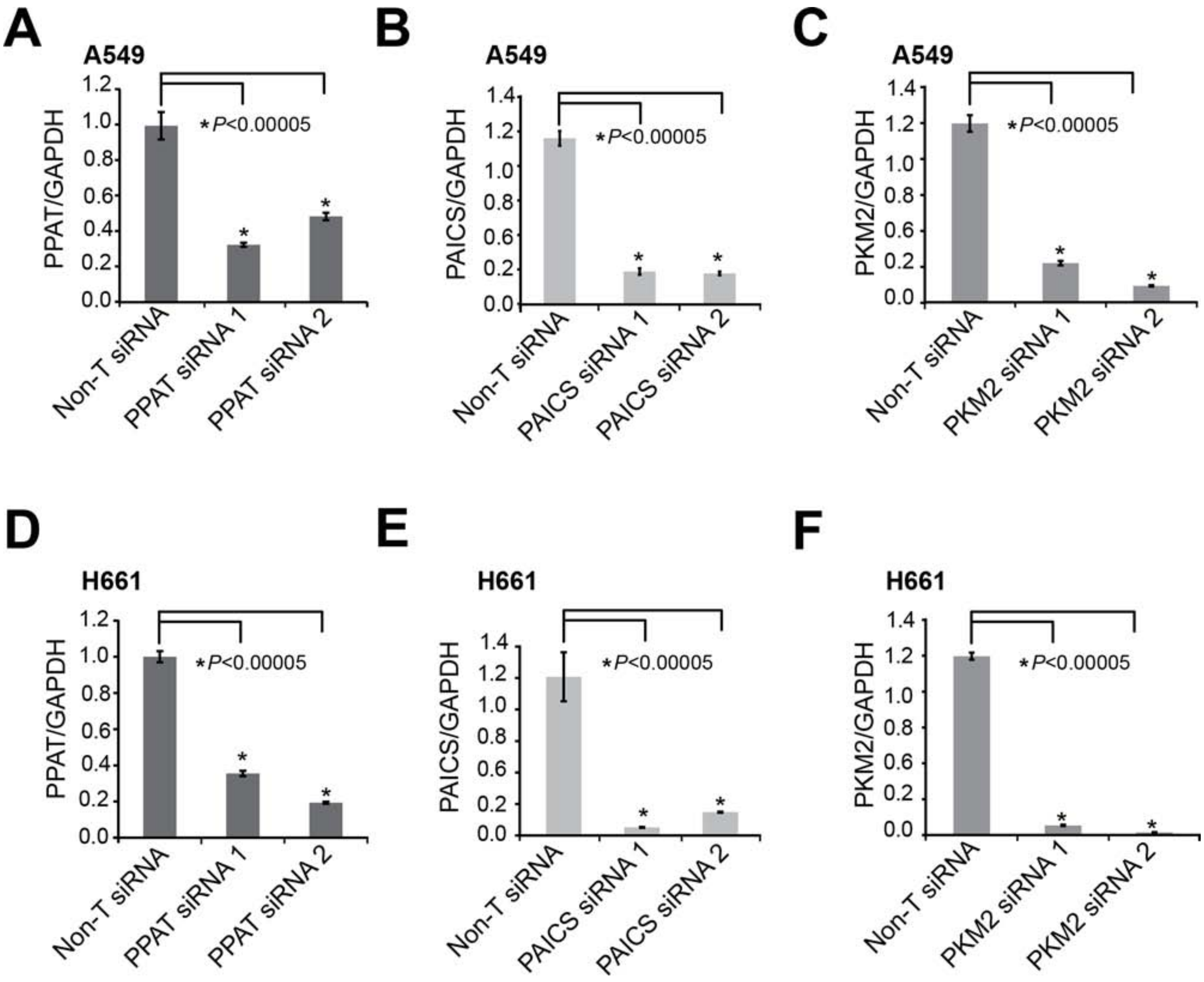

Supplementary Figure S4: RNA interference using siRNA duplex against *PPAT*, *PAICS* and *PKM2* decreases respective transcripts in multiple lung cancer cell lines. qRT-PCR analyses of **A, D.** *PPAT*, **B, E.** *PAICS* and **C, F.** *PKM2* transcripts levels in A549 and H661 cells using *GAPDH* for normalization of data. Two independent duplexes were used for the knockdown and measurement of transcript expression. Related to Figure 3.

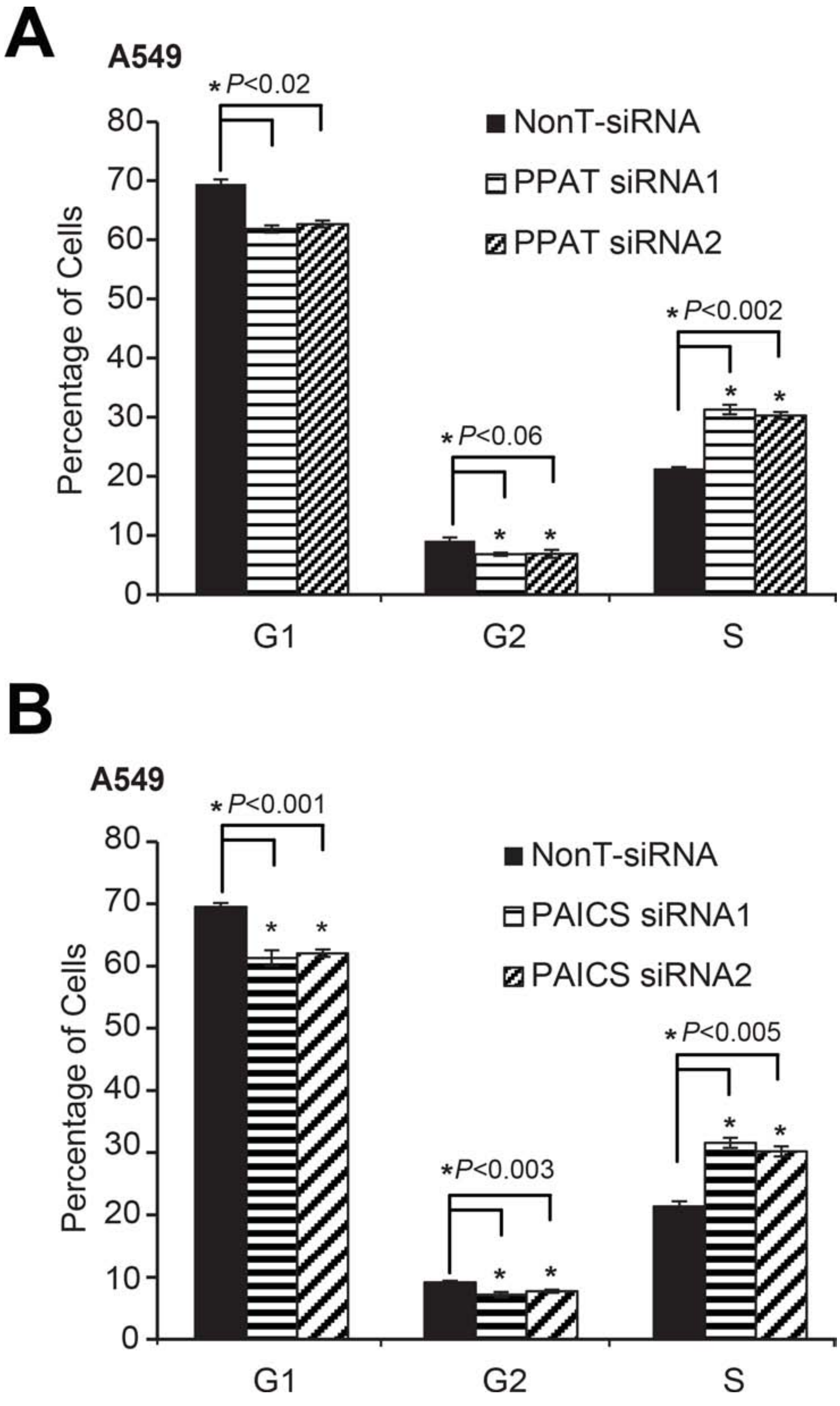

Supplementary Figure S5: Cell cycle analyses in *PPAT* and *PAICS* knockdown cells show arrest and increase in S-phase. Cell cycle analyses of A549 cells using siRNA against **A.** *PPAT* and **B.** PAICS genes respectively using two independent duplexes. Asterisk indicates data is statistically significant (*P* <0.05). Related to Figure 3.

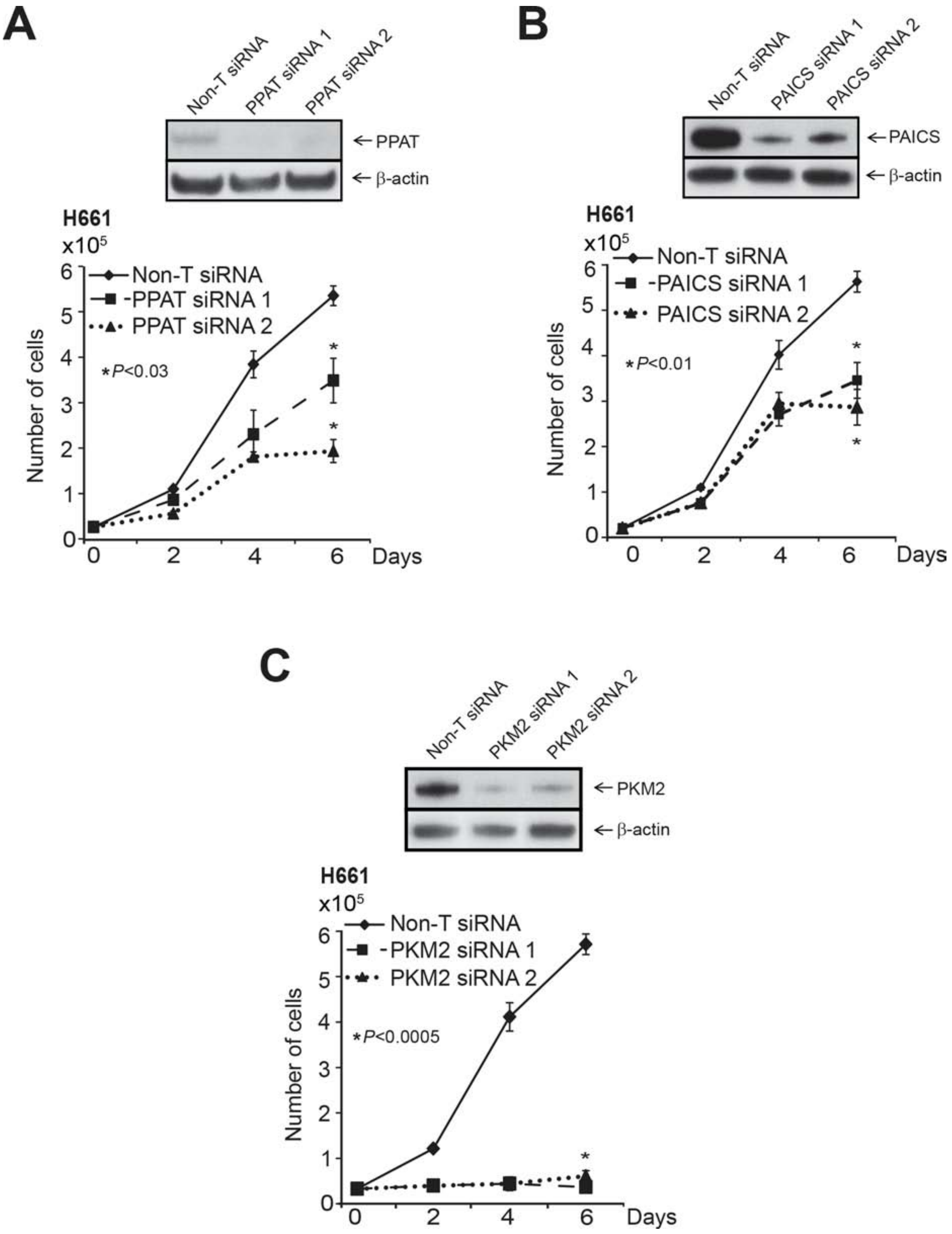

Supplementary Figure S6: RNA interference using specific siRNA duplex against *PPAT*, *PAICS* and *PKM2* decreases cell proliferation in H661, a large cell carcinoma cell line. **A.** *PPAT*, **B.** *PAICS* and **C.** *PKM2* knockdown was achieved using two independent duplexes followed by measurement of cell proliferation. The inset shows immunoblot analyses of the respective genes after transfection with the duplexes. β-actin was used as a loading control. Related to Figure 3.

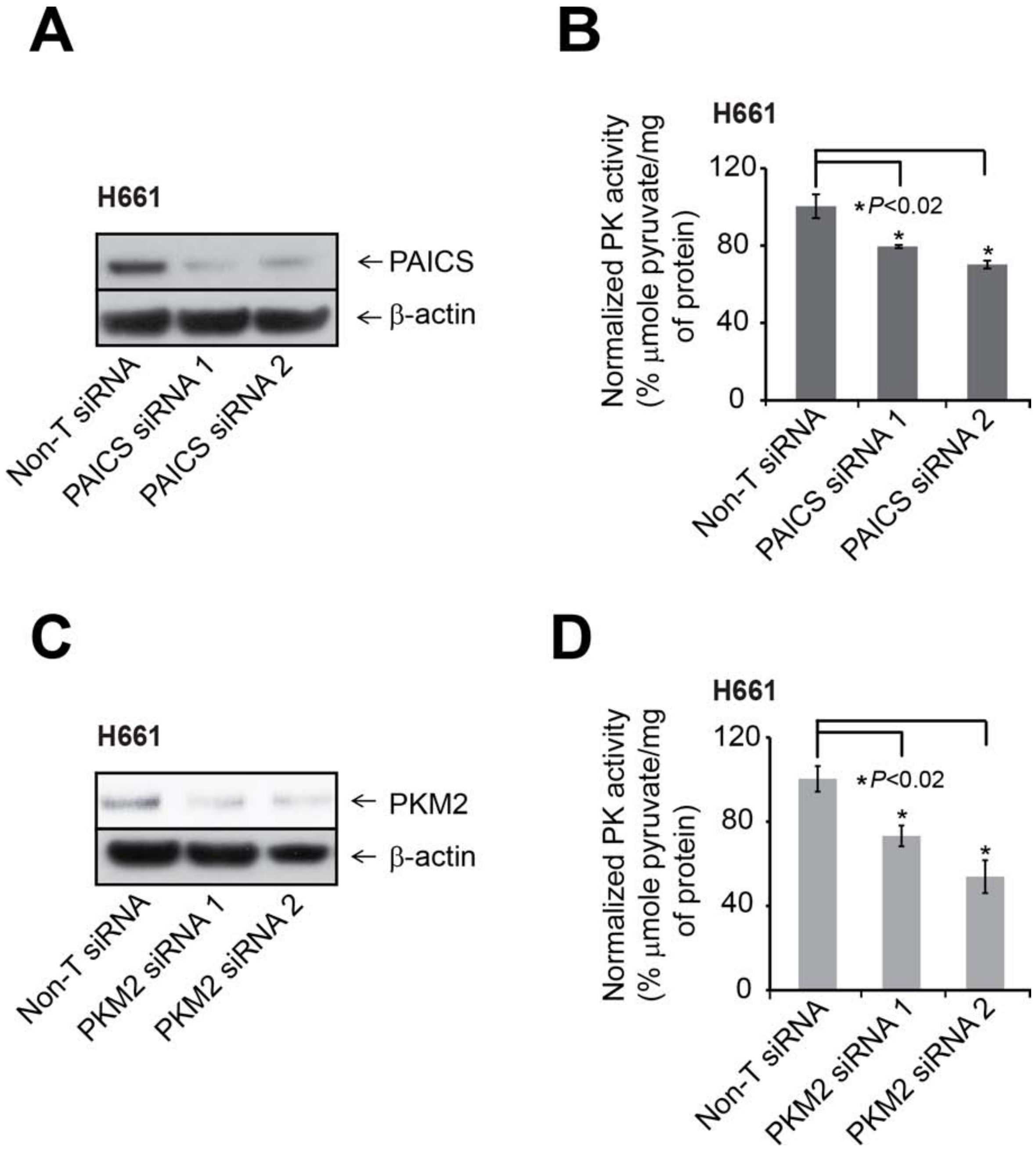

Supplementary Figure S7: PK activity is modulated on altering *PAICS* and *PKM2* levels in H661 cells. Knockdown of **A.** *PAICS* and **C.** *PKM2* was achieved using two independent duplexes tested using immunoblot analyses. β-actin was used as loading control. PK activity was measured in **B.** PAICS and **D.** *PKM2* knockdown cells. Related to Figure 4.

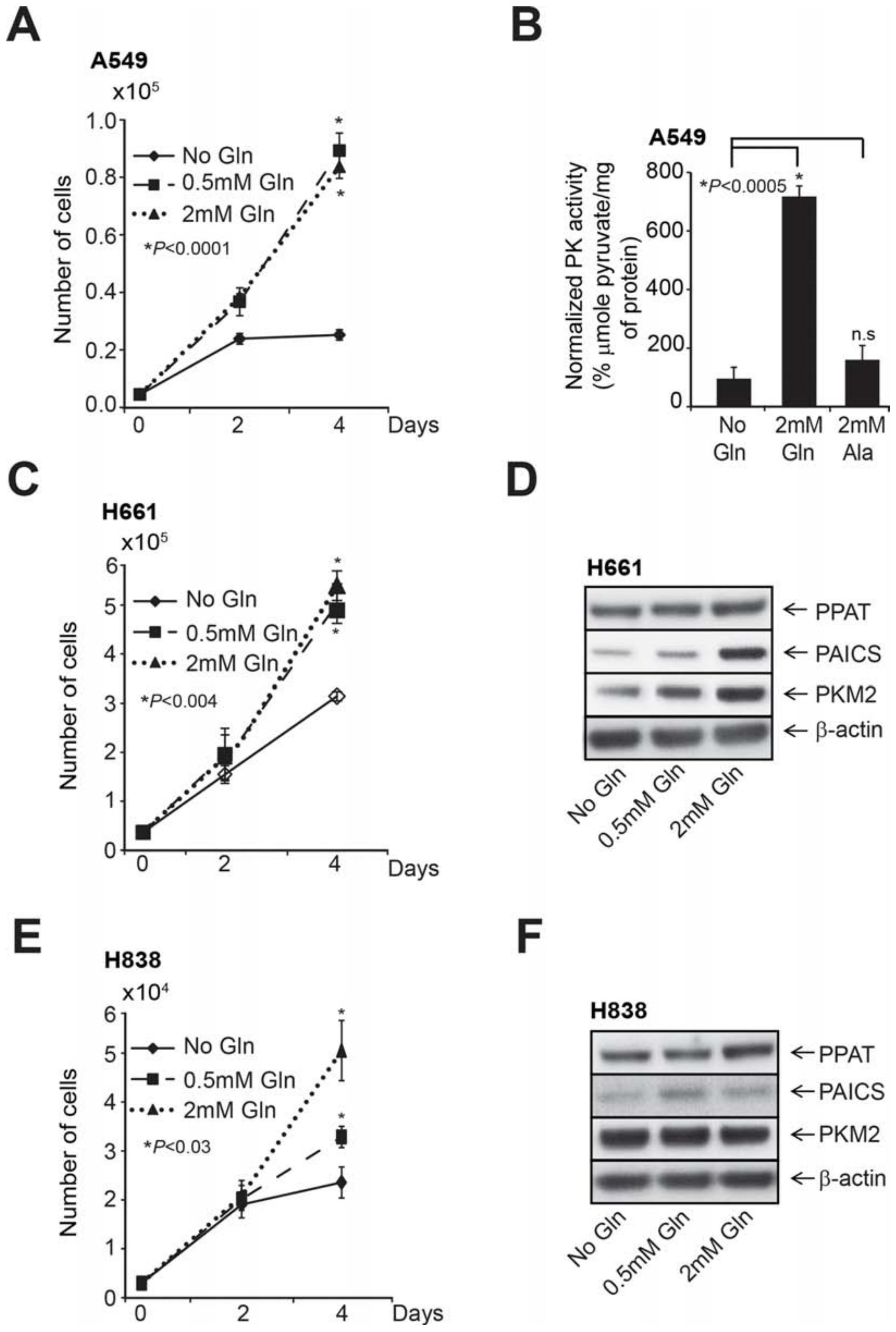

Supplementary Figure S8: Glutamine triggers cell proliferation and induction of *PPAT* and *PAICS* in multiple lung cancer cells. Glutamine triggered cell proliferation was measured in **A.** A549, **C.** H661 and **E.** H838 cells. **B.** PK activity was measured in presence of 2 mM glutamine and 2 mM alanine. Minus glutamine was used as a negative control. Glutamine induced *PPAT* and *PAICS* was measured in **D.** H661 and **F.** H838 using immunoblot analyses. β-actin was used as a loading control. Related to Figure 4.

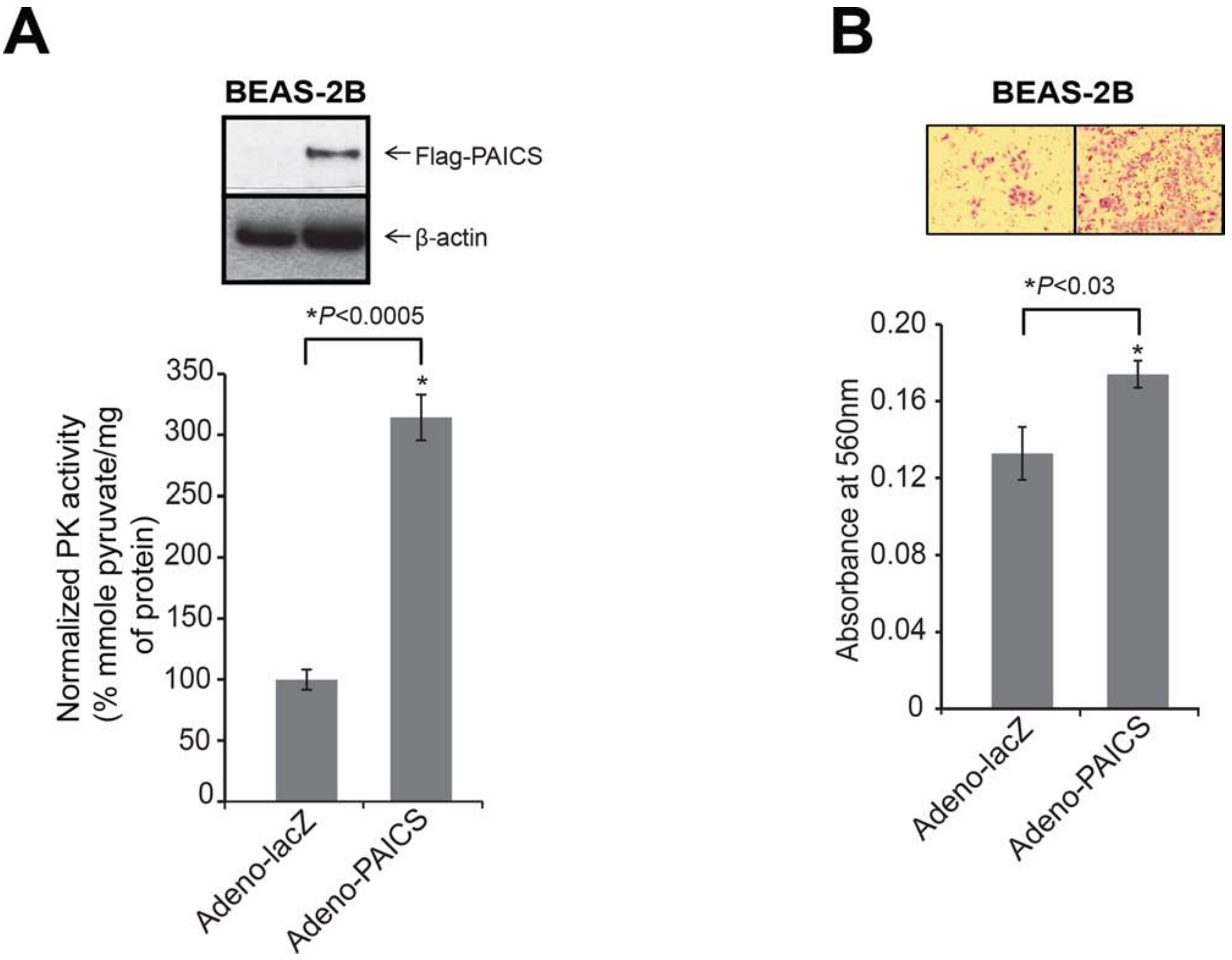

Supplementary Figure S9: *PAICS* overexpression induces pyruvate kinase activity and increased invasion in benign lung epithelial cells BEAS-2B. A. BEAS-2B cells were infected with adenovirus containing PAICS-flag (shown as inset immunoblot; flag-antibody#F1804, Sigma-Aldrich, USA) followed by measurement of PK activity. **B.** Following PAICS-Adenovirus transfections, BEAS-2B cell invasion was measured using Boyden Chamber Matrigel invasion assay. Related to Figure 4.

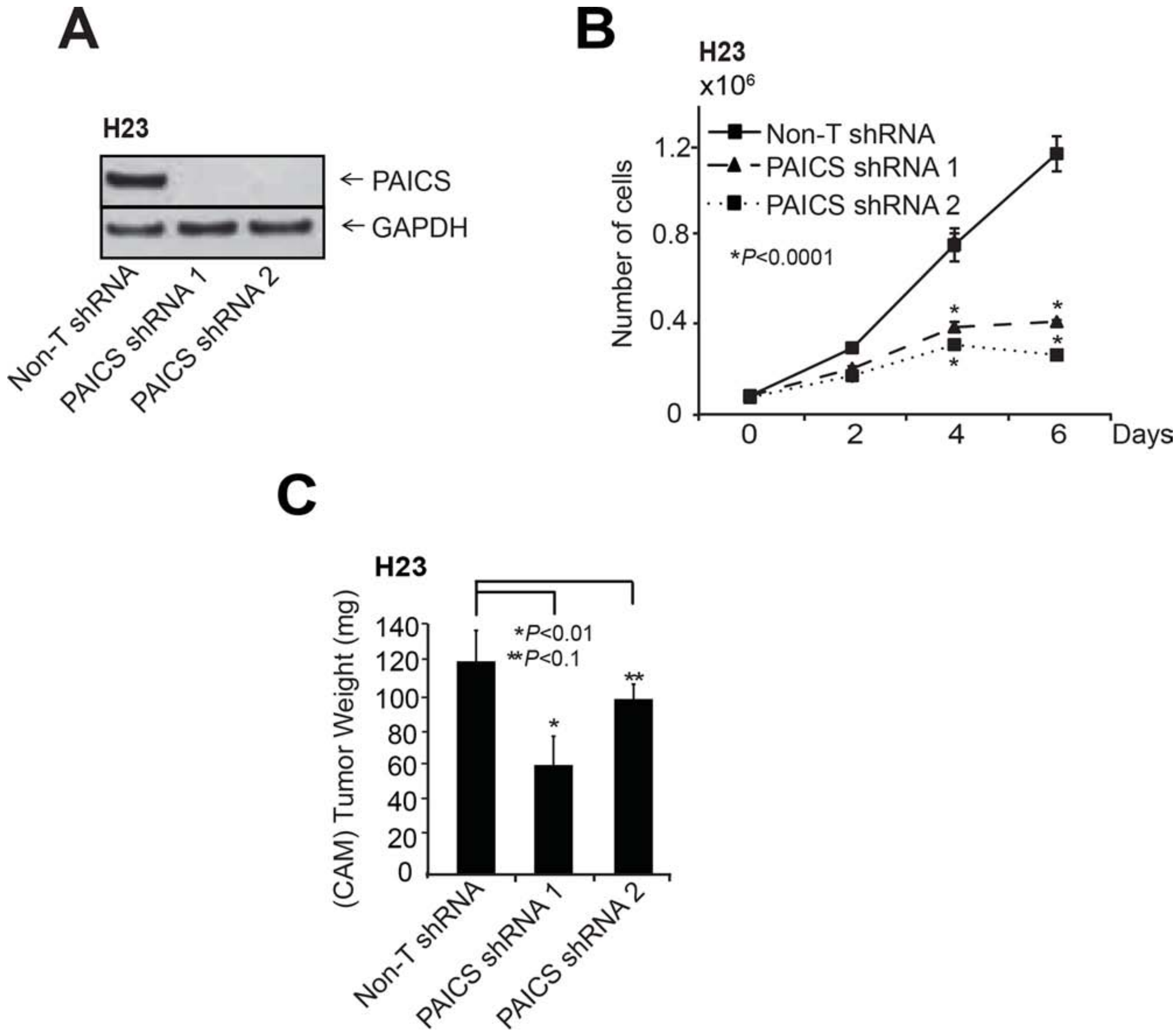

Supplementary Figure S10: Stable knockdown of *PAICS* in H23 decreases cell proliferation and tumor formation by CAM assay. Knockdown of *PAICS* in H23 was achieved **A.** by using *PAICS*-specific shRNAs and tested using immunoblot analyses followed by **B.** measuring cell proliferation. **C.** *PAICS* knockdown decreased tumor formation as evaluated by CAM assay. Related to Figure 5.

Supplementary Table S1. Correlation of PPAT, PAICS, PKM2 mRNA and clinical characteristics in 442 lung adenocarcinomas

Variable	Number	PKM2[*]	*P* value[**]
Age average (ys)	64.4		
<60	128	6.02	0.5
> = 70	149	6.13	
Gender			
Female	219	5.93	0.0001
Male	223	6.11	
N status			
N0	299	5.99	0.1
N1-2	141	6.07	
T status			
T1	150	5.93	
T2	251	6.03	0.03[+]
T3-4	39	6.29	0.0002[+]
Smoking			
Never	49	5.79	
Past	268	5.98	0.002[++]
Current	32	6.21	0.0004[++]
		**PAICS**[*]	***P* value**[**]
Age average (ys)	64.4		
<60	128	8.52	0.9
> = 70	149	8.64	
Gender			
Female	219	8.4	0.0002
Male	223	8.64	
N status			
N0	299	8,45	0.003
N1-2	141	8.66	
T status			
T1	150	8.44	
T2	251	8.52	0.2[+]
T3-4	39	8.82	0.001[+]
Smoking			
Never	49	8.17	
Past	268	8.47	0.007[++]
Current	32	8.85	0.0006[++]

(*Continued*)

Variable	Number	PKM2*	*P* value**
Age average (ys)	64.4		
<60	128	11.09	0.04
> = 70	149	10.87	
Gender			
Female	219	10.98	0.1
Male	223	11.05	
N status			
N0	299	10.97	0.005
N1-2	141	11.12	
T status			
T1	150	11	
T2	251	11.02	0.6+
T3-4	39	11.08	0.4+
Smoking			
Never	49	10.98	
Past	268	11.01	0.7++
Current	32	11.02	0.7++

AD, adenocarcinoma;
*mean value (data from Shedden, 2008 Nature Med, U133A Affymetrix, RMA normalized and log2 transformed);
***t* test;
+compared to T1;
++compared to Never

Supplementary Table S2. List of antibodies used in this study

Antibody	Application	Dilution	Supplier	Cat. No.
**PPAT**	IB	IB, 1:1000	Origene, Rockville, MD	TA504559
	IHC	IHC, 1:100		
**PAICS**	IB	IB, 1:10,000	Genetex, Irvine, CA	GTX83950
	IHC	IHC, 1:10000		
**PKM2**	IB (Tissue)	IB, 1:1000	Cell Signaling Technology	4053S
	IHC	IHC, 1:500	Danvers, MA	
	IB	IB, 1:1000	PTG lab, Chicago, IL	60268-1-Ig
**PKM1**	IB	IB, 1:1000	Cell Signaling Technology Danvers, MA	7067
**β -actin**	IB	IB, 1:20000	PTG lab, Chicago, IL	HRP-60008
**Total H3**	IB	IB, 1:1000	Cell Signaling Technology Danvers, MA	9715
**GAPDH**	IB	IB, 1:5000	PTG lab, Chicago, IL	HRP-60004
**Anti-mouse HRP**	IHC	Manufacturer instructions	Ventana Medical Systems Roche Diagnostics, USA	760-4313
**Anti-Rabbit HRP**	IHC	Manufacturer instructions	Ventana Medical Systems Roche Diagnostics, USA	760-4315

Supplementary Table S3. List and sequence of primers used in this study

Gene	Primers	Sequence	Reference
PAICS	Forward	GTGGCAGGCAGAAGTAATGG	Custom designed
PAICS	Reverse	CACATCCTGAACTCCCCAGT	Custom designed
PKM1	Forward	CGAGCCTCAAGTCACTCCAC	[43]
PKM1	Reverse	GTGAGCAGACCTGCCAGACT	[43]
PKM2	Forward	ATTATTTGAGGAACTCCGCCGCCT	[43]
PKM2	Reverse	ATTCCGGGTCACAGCAATGATGG	[43]
PPAT	Forward	GCGATTGAAGCACCTGTGGATG	Custom designed
PPAT	Reverse	CGGTTTTTACACAGCACCTCCAC	Custom designed
GAPDH	Forward	TGCACCACCAACTGCTTAGC	Custom designed
GAPDH	Reverse	GGCATGGACTGTGGTCATGAG	Custom designed

Supplementary Table S4. List and sequences of si and shRNAs used in this study

Gene	Sequence	Supplier	Cat. No.
PPAT si1	GAAAUGGUCUGGAAUGUUU	Dharmacon, CO, Thermo Fisher Scientific, Pittsburgh, PA	J-006003-06
si2	GGAAAUAUCCAGACACAAU	Dharmacon, CO, Thermo Fisher Scientific, Pittsburgh, PA	J-006003-07
PAICS si1	GUACACUGGUUGAUAUGAA	Dharmacon, CO, Thermo Fisher Scientific, Pittsburgh, PA	J-003980-07
si2	GAAGGGCUCCAAAUGGUAA	Dharmacon, CO, Thermo Fisher Scientific, Pittsburgh, PA	J-003980-09
PKM2 si1	AGGCAGAGGCUGCCAUCUA	Dharmacon, CO, Thermo Fisher Scientific, Pittsburgh, PA	Custom made
si2	CCAUAAUCGUCCUCACCAA	Dharmacon, CO, Thermo Fisher Scientific, Pittsburgh, PA	Custom made
PPAT sh1	TTGTAGGAATGTTTATTCC	Open Biosystems, Thermo Fisher Scientific, Pittsburgh, PA	V2LHS_170267
sh2	ATTCAAATGCCAATTTGCC	Open Biosystems, Thermo Fisher Scientific, Pittsburgh, PA	V2LHS_170269
PAICS sh1	GUACACUGGUUGAUAUGAA	System Biosciences, Mountain View, CA	Custom made
sh2	GAAGGGCUCCAAAUGGUAA	System Biosciences, Mountain View, CA	Custom made

REFERENCES

1. Selamat SA, Chung BS, Girard L, Zhang W, Zhang Y, Campan M, Siegmund KD, Koss MN, Hagen JA, Lam WL, Lam S, Gazdar AF, Laird-Offringa IA. Genome-scale analysis of DNA methylation in lung adenocarcinoma and integration with mRNA expression. Genome research. 2012; 22:1197–1211.
2. Seo JS, Ju YS, Lee WC, Shin JY, Lee JK, Bleazard T, Lee J, Jung YJ, Kim JO, Shin JY, Yu SB, Kim J, Lee ER, Kang CH, Park IK, Rhee H, et al. The transcriptional landscape and mutational profile of lung adenocarcinoma. Genome research. 2012; 22:2109–2119.
3. Cerami E, Gao J, Dogrusoz U, Gross BE, Sumer SO, Aksoy BA, Jacobsen A, Byrne CJ, Heuer ML, Larsson E, Antipin Y, Reva B, Goldberg AP, Sander C, Schultz N. The cBio cancer genomics portal: an open platform for exploring multidimensional cancer genomics data. Cancer discovery. 2012; 2:401–404.
4. Gao J, Aksoy BA, Dogrusoz U, Dresdner G, Gross B, Sumer SO, Sun Y, Jacobsen A, Sinha R, Larsson E, Cerami E, Sander C, Schultz N. Integrative analysis of complex cancer genomics and clinical profiles using the cBioPortal. Science signaling. 2013; 6:pl1.
5. Dhanasekaran SM, Alejandro Balbin O, Chen G, Nadal E, Kalyana-Sundaram S, Pan J, Veeneman B, Cao X, Malik R, Vats P, Wang R, Huang S, Zhong J, Jing X, Iyer M, Wu YM, et al. Transcriptome meta-analysis of lung cancer reveals recurrent aberrations in NRG1 and Hippo pathway genes. Nat Commun. 2014; 5:5893.
6. Shedden K, Taylor JM, Enkemann SA, Tsao MS, Yeatman TJ, Gerald WL, Eschrich S, Jurisica I, Giordano TJ, Misek DE, Chang AC, Zhu CQ, Strumpf D, Hanash S, Shepherd FA, Ding K, et al. Gene expression-based survival prediction in lung adenocarcinoma: a multi-site, blinded validation study. Nature medicine. 2008; 14:822–827.
7. Bild AH, Yao G, Chang JT, Wang Q, Potti A, Chasse D, Joshi MB, Harpole D, Lancaster JM, Berchuck A, Olson JA Jr, Marks JR, Dressman HK, West M, Nevins JR. Oncogenic pathway signatures in human cancers as a guide to targeted therapies. Nature. 2006; 439:353–357.
